# Individual connectivity-based parcellations reflect functional properties of human auditory cortex

**DOI:** 10.1162/imag_a_00486

**Published:** 2025-02-25

**Authors:** Maria Hakonen, Louisa Dahmani, Kaisu Lankinen, Jianxun Ren, Julianna Barbaro, Anna Blazejewska, Weigang Cui, Parker Kotlarz, Meiling Li, Jonathan R. Polimeni, Tori Turpin, Işıl Uluç, Danhong Wang, Hesheng Liu, Jyrki Ahveninen

**Affiliations:** Athinoula A. Martinos Center for Biomedical Imaging, Department of Radiology, Massachusetts General Hospital Charlestown, Boston, MA, United States; Department of Radiology, Harvard Medical School, Boston, MA, United States; Division of Brain Sciences, Changping Laboratory, Beijing, China; Harvard-MIT Program in Health Sciences and Technology, Massachusetts Institute of Technology, Cambridge, MA, United States; Biomedical Pioneering Innovation Center (BIOPIC), Peking University, Beijing, China

**Keywords:** auditory cortex, parcellation, functional connectivity, 7T fMRI, individual differences, human

## Abstract

Neuroimaging studies of the functional organization of human auditory cortex have focused on group-level analyses to identify tendencies that represent the typical brain. Here, we mapped auditory areas of the human superior temporal cortex (STC) in 30 participants (15 women) by combining functional network analysis and 1-mm isotropic resolution 7T functional magnetic resonance imaging (fMRI). Two resting-state fMRI sessions, and one or two auditory and audiovisual speech localizer sessions, were collected on 3–4 separate days. We generated a set of functional network-based parcellations from these data. Solutions with 4, 6, and 11 networks were selected for closer examination based on local maxima of the Dice coefficients and Silhouette values. The resulting parcellation of auditory cortices showed intraindividual reproducibility of 69–78% between resting-state sessions and 62–73% between resting-state and task sessions, indicating moderate reproducibility. The interindividual variability was significantly larger than intraindividual variability (Dice coefficient: 57%–68%, p < 0.001), indicating that the parcellations also captured meaningful interindividual variability. The individual-specific parcellations yielded the highest alignment with task response topographies, suggesting that individual variability in parcellations reflects individual variability in auditory function. Connectional homogeneity within networks was also highest for the individual-specific parcellations. Furthermore, the similarity in the functional parcellations was not explainable by the similarity of macroanatomical properties of the auditory cortex. Together, our results show that auditory areas in STC can be segmented into functional subareas based on functional connectivity. Our findings also suggest that individual-level parcellations capture meaningful idiosyncrasies in auditory cortex organization.

## 1 Introduction

Differences in behavior and cognition in individual people have been shown to reflect individual-specific variability of patterns of functional connectivity (Baldassarre et al., 2012;Briley et al., 2022;Finn & Todd Constable, 2016;Godefroy et al., 2022;Ranasinghe et al., 2014;Rosenberg et al., 2020;Weissberger et al., 2020;Wig et al., 2014;Yang et al., 2021). Individual variability may also be one of the major factors underlying why a widely accepted model of the human auditory cortex (AC), analogous to that found in non-human primates (Hackett et al., 1999;Kaas & Hackett, 2000;Kusmierek & Rauschecker, 2009;Rauschecker et al., 1995), is still lacking (Moerel et al., 2021). Microanatomical and functional neuroimaging studies provide evidence that the human AC can be divided into core, belt, and parabelt areas, as well as into parallel anterior versus posterior feature pathways, which resemble the subdivisions of non-human primate models (Ahveninen et al., 2014;Alain et al., 2001;Kaas & Hackett, 2000;Moerel et al., 2014;Rauschecker & Tian, 2000;Schönwiesner et al., 2015). However, contrasting interpretations exist on the exact layout of these broader arrangements in humans.

In recent years, the development of ultra-high-resolution functional magnetic resonance imaging (fMRI) at 7 tesla (7T) has allowed a deeper investigation into the tonotopy of the primary AC (Da Costa et al., 2011,^2015^;Formisano et al., 2003;Talavage et al., 2004) and the superior temporal plane (Ahveninen et al., 2016;Langers & van Dijk, 2012;Moerel et al., 2014;Striem-Amit et al., 2011;Talavage et al., 2004). 7T fMRI allows higher spatial resolution without compromising the signal-to-noise ratio (Uğurbil, Adriany, et al., 2003). Additionally, at 7T, the fMRI signal reflects more closely underlying neuronal activity since the contribution of smaller vessels to the fMRI signal increases with the field strength (Ogawa et al., 1992;Uğurbil, Toth, et al., 2003). The small voxel size also reduces partial volume effects (Newton et al., 2012). 7T fMRI studies have provided converging evidence that there is a large area on Heschl’s gyrus (HG) that is sensitive to low frequencies and is surrounded posteriorly, antero-medially, and antero-laterally by areas sensitive to high frequencies. Interestingly, an individual-level analysis suggested that the AC contains additional frequency reversals that were not evident in the group-level maps of the previous studies (De Martino et al., 2015;Moerel et al., 2013,^2014^).

While the other sensory cortices are relatively similar across individuals, the human AC shows larger interindividual anatomical and functional variability (Hackett et al., 2001;Moerel et al., 2013,^2014^;Ren, Xu, et al., 2021), a feature that is typical of associative brain areas. AC also resembles associative areas in its anatomy and function, as it includes integrative connections (Lu & Wang, 2004), both early and late ACs have several connections to the prefrontal cortex (Plakke & Romanski, 2014), and AC has been shown to integrate information from different senses as a multimodal representation (Eggerrmont, 2015). Auditory processing is also strongly modified by attention (Ahveninen et al., 2011;Jääskeläinen & Ahveninen, 2014;Laffere et al., 2020;Lage-Castellanos et al., 2022). Moreover, AC is highly plastic, and its function is shaped by experience (for a review seeAlkhateeb et al., 2019andIrvine, 2018), which further underlines its interindividual variability. Indeed, AC seems to be an essential part of the networks of brain regions responsible for higher-order cognitive processes, such as language processing (Hertrich et al., 2020), working memory (Ahveninen et al., 2023;Kumar et al., 2016;Mamashli et al., 2021;Uluc et al., 2018), auditory perceptual decision-making, learning, and prediction (King et al., 2018). While cortical parcellations have conventionally been generated by spatial normalization and averaging the data over several individuals to increase the signal-to-noise ratio, this approach may not be appropriate for revealing the fine, unique subregions of the human AC since it blurs potentially meaningful interindividual variation.

Parcellation approaches relying on individual-specific functional connectivity provide an interesting opportunity to capture the unique functional organizations of individual brains complementing previous studies that have typically defined the regions of interest based on anatomy or population-averaged functional data. Individual-level functional mapping can also enhance group-level analyses since it allows aligning data across participants according to their functional characteristics instead of anatomical landmarks (Wang & Liu, 2014). Indeed, individual-level parcellations have become a very active topic of investigation and a variety of functional connectivity-based approaches have been used to uncover individual differences in the functional organization of the human cortex (Chen et al., 2015;Gordon et al., 2017;Jakobsen et al., 2018;Salehi et al., 2018;Wang et al., 2015;Zhang et al., 2021;Zhao et al., 2020). However, most of the human AC parcellations are still derived from functional specifications (e.g., tonotopic mapping) and microanatomical architecture (e.g., cyto-, myelo-, or receptor architecture; for a review, seeMoerel et al., 2014). A recent study investigated subdivisions of Heschl’s gyrus (HG) in individuals using a parcellation approach that relied on structural connectivity derived from diffusion MRI tractography (Lee et al., 2022). However, detailed functional connectivity-based parcellations in individuals are still needed.

**Fig. 1.**
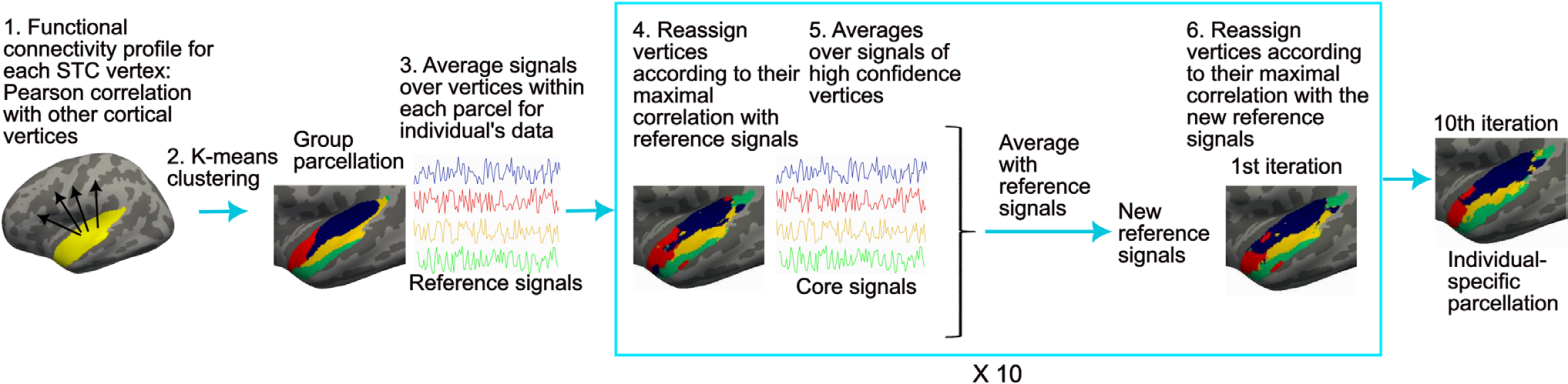
Individual-level functional parcellation method. (1) Pearson correlation was computed between the fMRI signal at each STC vertex and signals of all other cortical vertices in each participant. The individual-level connectivity profiles were averaged across participants at each vertex using a cortical surface-based atlas. (2) Group-level parcellation was computed by using K-means clustering, to cluster vertices with similar connectivity profiles into the same network. (3) The individual participant’s fMRI signal time courses were then averaged across the vertices within each group-level network (reference signals). (4) The parcellation was adapted for each individual participant by reassigning vertices to networks according to their maximal correlation with the reference signals. For each vertex, a confidence value was computed as the ratio of the largest and second largest correlations. (5) For each new network of the adapted parcellation, an average was computed using the connectivity profiles of the vertices with confidence values larger than 1.3. The resulting “high-confidence signals” were averaged with the reference signals. (6) Using the resulting new reference signals, the STC vertices were further reassigned to one of the networks. Steps 4–6 were repeated 10 times to generate an individual-specific parcellation. Ten iterations were selected because the parcellations started to be stable at the 5th iteration as measured by The Dice coefficient.

Here, we investigated functional subareas of the human auditory cortex in the superior temporal cortex (STC) in individuals using 1-mm resolution 7T fMRI and a cortical parcellation method that is based on functional connectivity (Wang et al., 2015;Fig. 1). The parcellation method identifies functional networks in individuals by iteratively adjusting a population-based functional atlas to fit each individual’s idiosyncratic features. This method has been shown to be able to generate highly reproducible parcellations of the whole cortex within individuals and capture meaningful interindividual differences (Wang et al., 2015). Because the higher field strength decreases partial volume artifacts (Newton et al., 2012) and increases the relative contribution of small vessels compared to large draining veins (Ogawa et al., 1992;Uğurbil, Toth, et al., 2003), it can be expected that the individual variability of small auditory areas also reflects more closely variability in the brain function rather than in the vasculature. 7T has also demonstrated higher correlation coefficients between connected brain regions, indicating its greater sensitivity to temporal correlation (Hale et al., 2010;Kreitz et al., 2023). To validate the test-retest reliability of the results in this study, we measured fMRI data from 30 participants during two resting-state sessions and 1–2 auditory and audiovisual stimulation sessions. Each session was conducted on a different day. We hypothesized that STC can be divided into functional subareas that show high intraindividual reproducibility between sessions, and that the topography and functional specialization of these subareas reveal substantial interindividual variability.

## 2 Methods

### 2.1 Participants

Thirty healthy volunteers (32.4 ± 10 years, 15 women, all right-handed) were recruited in this study via the Rally website that advertises internal clinical research studies of Massachusetts General Brigham (MGB). According to our bootstrapping-based power calculations (Kleinman & Huang, 2016), even after conservative post-hoc correction procedures, with this sample size we achieved a statistical power of 0.9 (Fig. S1).

Twenty-eight of the participants were native English-speakers. None of the participants reported the use of cognition-altering medications, difficulties in hearing, or exposure to excessive noise. The study protocol was approved by the Institutional Review Board at MGB. The study was carried out in accordance with the guidelines of the declaration of Helsinki. A written informed consent was obtained from all participants prior to participation. Twenty of the participants participated in an additional hearing test. Based on Hughson-Westlake pure tone procedure, all but one participant had a hearing threshold of under 25 dB at 0.125–8 kHz. One of the participants had a 40-dB threshold at the highest frequencies of 6 and 8 kHz in the left ear and 35 dB at the frequency of 8 kHz in the right ear. Because of our goal to develop and test an individualized functional parcellation strategy for wide purposes, this participant was included in the study. Further, similarly to all other participants, this participant performed within a normal range in a computerized, automatic speech-in-noise comprehension task (QuickSIN test procedure; SNR loss: mean: –0.44, standard error of the mean: 0.2, range: –1.8–0.9). The supplemental hearing tests were conducted using a Diagnostic Audiometer AD 629 (Interacoustics, Middelfart, Denmark) with IP30 insert earphones.

### 2.2 Experimental protocol

Twenty-two participants completed three fMRI sessions on different days, and eight completed them in 4 days. Two of the sessions included six 8-min resting-state fMRI scans (for one participant, only four scans were performed in one resting-state session). The reproducibility of functional connectivity has shown marked improvement up to 27 min of 3T fMRI data, with additional benefits observed up to 90–100 min of data collection (Laumann et al., 2015). Additionally, 7T fMRI has demonstrated greater sensitivity in detecting functional connectivity compared to 3T fMRI (Hale et al., 2010;Kreitz et al., 2023). Resting-state functional connectivity has been widely used to estimate brain functional network parcellations (Kong et al., 2021;Laumann et al., 2015;Salehi et al., 2018;Schaefer et al., 2018;Wang et al., 2015;Wig et al., 2014;Yan et al., 2023;Yeo et al., 2011). It reflects intrinsic connectivity and brain functional organization in a task-free context. The additional tasks, which were performed in up to two separate sessions, included a combined tonotopy and amplitude modulation (AM) rate representation task (2 × 8 min) and an audiovisual speech perception task (4 × 11 min). The tasks were used to validate data quality and estimate functional specificity of the parcellations. The duration of each session, with preparation, was around 2 h. The participants were instructed to keep their eyes open and avoid any movement during all scans. Foam paddings were used to attenuate head movements and scanner noise. The hearing of the participants was also protected with earplugs in the resting-state scans. In the task sessions, the auditory stimuli were delivered to the participants’ ears through MR-compatible insertable earphones (Sensimetrics Corporation, Model S15, Woburn, MA, USA) that were designed to effectively attenuate the scanner noise. During the resting-state scans, the participants were presented with a white fixation cross on a black background. In the task sessions, participants were instructed to attentively perform the given auditory task by indicating their responses through the use of an MRI-compatible response pad.

The “Tonotopy/AM task” was used to localize tonotopy and amplitude modulation rate gradients. The participants were presented with serial blocks consisting of seven sounds, which varied both in their center frequency (tonotopy mapping) and in their AM rate (rate representation mapping). The presentation order of the blocks was optimized using an automatic event scheduling software, Optseq (FreeSurfer:https://surfer.nmr.mgh.harvard.edu/optseq/), and the presentation order of sounds within the blocks was randomized using randperm (MATLAB). The carrier sounds were octave-wide bands of filtered white noise whose center frequencies were 0.12, 0.47, 1.87, or 7.47 kHz. Broadly filtered white noise was used to investigate tonotopic subareas both within and outside of the core auditory areas. The general idea of the present task was adapted from studies, which suggest that using complex tasks and stimuli can enhance the mapping of feature-topographic representations even in the higher-order cortices (Bressler & Silver, 2010;Saygin & Sereno, 2008). These four possible carrier signals were modulated at rates centered either at around 4 Hz (*slow AM*) or at around 32 Hz (*fast AM*). In 85% of these blocks, which were non-targets, the AM rate was varied randomly at seven possible 1/8 octave steps around, and including, the center of the AM rate window, akin to an “AM melody” (adapted and modified fromNorman-Haignere et al., 2019). In the remaining 15%, which were the target blocks, the AM rate was either 4 or 32 Hz. Participants were asked to look at a fixation mark at the screen, ignore the changes in the carrier frequency (i.e., the tonotopy aspect), pay attention to the AM rate of the sounds, and press a button with their right index finger upon hearing a target block with a constant AM rate. Each 8-min run included 64 sound blocks, interleaved with silent baseline blocks.

The “Audiovisual Speech/Noise task” was used to localize brain areas processing speech and audiovisual information. The participants were presented with 636 (159 in each run) audiovisual recordings of a female talker speaking the words “rain” or “rock” (Ozker et al., 2017,^2018^). The auditory component was either acoustically intact or replaced with noise that matched the spectrotemporal power distribution of the original speech (for details of the stimulus creation, seeOzker et al., 2017). Similarly, the visual component was either intact or a blurred counterpart of the original version. The two types of auditory and visual components were combined, resulting in four conditions for both “rain” and “rock”: (1) Auditory Clear/Visual Clear, (2) Auditory Clear/ Visual Noisy, (3) Auditory Noisy/Visual Clear, and (4) Auditory Noisy/Visual Noisy. The stimuli were downloaded fromhttps://openwetware.org/wiki/Beauchamp:Stimuli#Stimuli_from_Ozker_et_al.Participants were asked to press a button with their right index finger when hearing “rain” and another button with their right middle finger upon hearing “rock.”

The stimulus presentation was controlled with Presentation software (Neurobehavioral Systems, Berkeley, CA, USA). The intensity level of the stimuli was adjusted individually at a comfortable listening level that was clearly audible above the scanner noise. The fixation cross and the visual stimuli were projected to a mirror mounted on the head coil.

### 2.3 Data acquisition

The brain imaging data were acquired using a 7T whole-body MRI scanner (MAGNETOM Terra, Siemens, Erlangen, Germany) and a home-built 64-channel receive brain array coil and a single-channel birdcage transmit coil (Mareyam et al., 2020). To minimize head motion, participants’ heads were stabilized with MRI-compatible foam pads. In each scanning session, T1-weighted anatomical images were collected using a 0.75-mm isotropic Multi-Echo MPRAGE pulse sequence (van der Kouwe et al., 2008; repetition time, TR = 2530 ms; echo time, 4 echoes with TEs 1.72, 3.53, 5.34 and 7.15 ms; flip angle = 7°; field of view, FoV = 240 × 240 mm^2^; 224 sagittal slices). To improve the accuracy of pial surface reconstruction, T2-weighted anatomical images were additionally acquired for 28 out of 30 participants with the T2 SPACE sequence (voxel size = 0.83 × 0.83 × 0.80 mm, TR = 9000 ms, TE = 269 ms, flip angle = 120°, FoV = 225 × 225 mm^2^, 270 sagittal slices) in one session.

Blood-oxygenation-level-dependent (BOLD) functional MRI data were collected using a single-shot gradient-echo blipped-CAIPI simultaneous multi-slice (SMS, acceleration factor in slice-encoding direction: 3; acceleration factor in phase-encoding direction: 4; 2D echo planar imaging (EPI) sequence;Setsompop et al., 2012); TR = 2800 ms; TE = 27.0 ms; isotropic 1-mm^3^voxels; flip angle = 78°; FoV = 192 × 192 mm^2^; 132 axial slices; phase enc. dir.: anterior→posterior; readout bandwidth = 1446 Hz/pixel; nominal echo spacing = 0.82 ms; fat suppression). For de-warping of the functional data, each session included an EPI scan collected with the same parameters, but in an opposite phase encoding polarity (posterior→anterior, PA-EPI) and a gradient-echo B0 field map (TR: 1040 ms, TEs: 4.71 ms and 5.73 ms; isotropic 1.3-mm^3^voxels; flip angle: 75°; FoV: 240 × 240 mm^2^; 120 slices; bandwidth = 303 Hz/pixel).

During the fMRI data acquisition, participants’ heart rate and respiration signals were recorded at 400 samples/s. The heart rate was measured using the built-in Siemens photoplethysmogram transducers placed on the palmar surface of the participant’s index finger. Respiratory movements were recorded with the built-in Siemens respiratory-effort transducer attached to a respiratory belt, which was placed around the participant’s chest.

### 2.4 Data preprocessing

The bias field of the anatomical T1 and T2 images was first corrected using SPM12 (http://www.fil.ion.ucl.ac.uk/spm/, spm_preproc_run.m; full-width at half-maximum, FWHM: 18 mm, sampling distance: 2 mm, bias regularization: 1e-4) and custom MATLAB scripts. Thereafter, the cortical reconstructions were generated for each participant using recon-all function of FreeSurfer 7.1 (Fischl, 2012) with an extension for submillimeter 7T data (Zaretskaya et al., 2018). Both the T2 image and an average over 3–4 T1 images from different sessions were used in the reconstruction to improve the quality of the cortical surfaces. For intracortical smoothing, nine additional surfaces were generated at fixed relative distances between the white and pial surfaces produced by recon-all. Finally, the quality of the reconstructed surface was reviewed for quality assurance, and pial and white mater segmentation errors were corrected using the Recon Edit function of Freeview.

fMRI analysis was conducted using FreeSurfer 7.1. The fMRI data were first slice-time and motion corrected. Geometric distortions induced by inhomogeneities in the static magnetic field were compensated by dewarping. The distortion field for dewarping was estimated based on the fMRI data and the PA-EPI scan which have equal and opposite distortions compared to the AP-EPI scans used for BOLD fMRI acquisition. From these scans, the susceptibility-induced off-resonance field offsets were estimated using a method described inAndersson et al. (2003)as implemented in the FMRIB Software Library (FSL;Smith et al., 2004; topup, applytopup, FSL 5.0.7). For four participants with a missing PA-EPI scan, the distortion field was estimated using the B0 field map (epidewarp, FreeSurfer 7.1). Heart rate and respiratory artifacts were corrected with RETROspective Image CORrection (RETROICOR) algorithm (Glover et al., 2000; 3rd-order heart rate, respiratory, and multiplicative terms). For the three participants with missing heart rate recordings, only respiratory data were used in RETROICOR. RETROICOR was not applied on five participants’ data because of missing heart rate and respiration recordings. Boundary-based registration (Greve & Fischl, 2009; preproc-sess, FreeSurfer 7.1) was used to co-register the functional data with the anatomical images. The fMRI data were smoothed spatially using an intracortical smoothing procedure applied within the cortical gray matter to reduce noise contamination and signal dilution from surrounding cerebrospinal fluid and white matter, resulting in higher gray matter specificity compared to conventional three-dimensional volume-based smoothing (Andrade et al., 2001;Blazejewska et al., 2019;Jo et al., 2007;Kiebel et al., 2000). For smoothing, the fMRI time series were resampled onto the 11 intermediate intracortical surfaces by projecting each intersecting voxel onto the corresponding surface vertices using trilinear interpolation. These surfaces were used as an input for the smoothing algorithm that generated one intracortically smoothed surface. The data were smoothed using a three-dimensional kernel that smooths tangentially with the 7th-order vertex neighborhood, and radially across 10 cortical surfaces. The top surface of the gray matter was excluded to reduce partial volume effects from the surrounding cerebrospinal fluid. Thereafter, the data were bandpass filtered between 0.01–0.08 Hz and signals from white matter, cerebrospinal fluid, and the area outside of the head were regressed out. The last two steps were performed only for the parcellation but not for GLM analysis since they may decrease the effect of interest.

### 2.5 Data analysis

#### 2.5.1 Group-based functional atlas

The individual-level parcellation algorithm was initialized with a group-level functional network atlas estimated for the auditory areas using the same approach as inRen, Hubbard, et al. (2021). The group-level atlas was used to mitigate the contribution of individual-specific noise signals (e.g., physiological, motion) to network mapping, and to capture general principles of AC functional organization. The group-level parcellation was generated in the FreeSurfer average subject space (fsaverage6, 40,962 vertices per hemisphere). The fMRI time-series data from each individual subject was projected into this surface space, creating time-series data with 81,924 vertices. The STC area was defined by combining superior temporal and transverse temporal labels from Desikan-Killiany atlas (Desikan et al., 2006). These areas were selected based on previous studies of auditory cortex anatomy and function (Kepinska et al., 2023;Worschech et al., 2022;Zachlod et al., 2020). For each STC vertex of every participant, its functional connectivity profile was estimated by computing the Pearson correlation between its time series and that of each of the other cortical vertices (including both hemispheres), that is, each functional connectivity profile was an 81,923 × 1 vector of correlation coefficient values. Thereafter, the functional connectivity profiles of each STC vertex were averaged across participants, and a k-means clustering algorithm was applied to cluster the vertices into networks based on the cosine similarity of their functional connectivity profiles. To avoid getting trapped in local minima, the clustering was performed 500 times with randomly selected initial network centroids, and the clustering solution was selected that yielded the smallest aggregate distance. We varied the network number from 2 to 24 and selected for individual-level investigation four solutions that yielded local maxima of the product of their Dice and Silhouette coefficients (4-, 6-, 11- and 19-network parcellations;Fig. S2; 19-network parcellation was excluded from the main analyses since it was not reproducible at the individual level). To compute the Dice coefficients for this analysis, the fMRI data from each run were split in half, the first halves were concatenated together, and the last halves were concatenated together. This allows better estimation of the reliability of the algorithm since differences in the head positioning between sessions do not affect the results. However, for the other analyses, all Dice coefficients were computed between parcellations derived from different sessions to make the Dice coefficients more comparable within versus between individuals. Thereafter, the Dice coefficients were computed between the two parcellations generated from these concatenated data. The Dice coefficient represents the percentage of vertices that were assigned into the same network in separate parcellations. The Dice coefficient was computed for each network in the parcellation as follows:

$$Dice\;coefficient\;=\frac{2\times V_{overlap}}{V_{1}+V_{2}}$$

where$V_{overlap}$is the number of vertices assigned into the same network in the parcellations, and$V_{1}$and$V_{2}\;$are numbers of vertices of the network in each of the parcellations. The Silhouette value was used to measure how similar the functional connectivity profile of a vertex is to the other connectivity profiles in the same cluster compared to the connectivity profiles of the other clusters. The Silhouette coefficient was calculated as follows:

$$Silhouette\;score=\;\frac{b-a}{\text{max}\left(a,b\right)}$$

whereais an average distance between each data point within a cluster andbis the average distance between all clusters.

Clusters of size 10 mm^2^or smaller were reassigned to larger clusters. To this end, the network assignment was computed for six neighboring vertices for each vertex within these small clusters. Thereafter, each vertex was assigned to the network that was the most frequent among the neighboring vertices. If there were equally frequent networks among the neighbors, a Pearson correlation was computed between the functional connectivity profile of the vertex and the average functional connectivity profile of the most frequent networks. The vertex was assigned to the network with which the correlation was highest. This procedure resulted in reassignment of one vertex in the left hemispheric 4-network parcellation and 5 vertices in the left hemispheric 11-network parcellation.

#### 2.5.2 Individual-level parcellation

Individual-level parcellations were generated by adjusting the functional connectivity-based group parcellation using the same procedure as used byWang et al. (2015). (1) Individual participant’s fMRI data were projected into the fsaverage6 surface (FreeSurfer, mri_surf2surf), and fMRI signal time courses were averaged across the vertices that fell within each network of the group-level functional network parcellation. These network time courses were used as the “reference signals” for the following individual-level adjustment procedure. (2) Each vertex of an individual participant was reassigned to one of the networks according to its maximal correlation with the corresponding reference signals. A confidence level was computed for each reassignment by dividing the strongest correlation value by the second strongest one. (3) A “high-confidence signal” was created for each network by averaging all vertices that were reassigned to that network that exceeded a confidence value of 1.3. (4) The high-confidence and original reference signals derived from the group parcellation were averaged, and the resulting functional connectivity profiles were used as new reference signals for the next iteration. This allowed us to use both individual participant’s information and the information of the group parcellation to guide the reassignment of the vertices. Finally, steps 3–4 were iterated 10 times, and the end result was used as the individual parcellation. Ten iterations were used since the parcellations start to became stable approximately at the 5th iteration (Dice coefficients of resting-state parcellations between 9th and 10th iterations: left: 4 networks: 99 ± 1%, 6 networks: 99 ± 1%, 11 networks: 97 ± 3%; right: 4 networks: 99 ± 0%, 6 networks: 99 ± 1%, 11 networks: 97 ± 4%), and a high number of iterations increases the risk of overfitting the data. Individual-level parcellations were generated for 4-, 6-, and 11-network group parcellations using the fMRI data from (1) the first resting-state session, (2) second resting-state session, (3) task session, and (4) all sessions. The reproducibility of the parcellations was estimated by comparing the parcellations generated based on different sessions.

We rejected one network from the left and one from the right hemisphere of the individual-level 11-network parcellations since they were not reproducible. Moreover, they did not appear to be neurophysiologically plausible since they included clusters of very few vertices distributed across the STS. The corresponding contralateral networks were reproducible and, therefore, they were not rejected. These networks were also unilateral in the 11-network group parcellation. The other networks were bilateral both in the individual and group parcellations.

#### 2.5.3 Intraparticipant reproducibility and interindividual variability of the parcellations

Intraparticipant reproducibility and interindividual variability of the parcellations were estimated with the Dice coefficient. The Dice coefficient for the parcellation as a whole was computed by averaging the network-specific Dice coefficients. To estimate the reproducibility of the parcellations, the Dice coefficients were computed between the parcellations derived from the two resting-state sessions collected on different days. We also tested whether the parcellation algorithm can generalize to different tasks by computing the Dice coefficients between the parcellations generated based on resting-state and task fMRI data. The interindividual variability was determined by averaging the Dice coefficients computed between each participant and all other participants within the resting-state sessions. Further, we compared the Dice coefficients between task and resting-state parcellations of the same participant with the Dice coefficients between task parcellations of the participant and any other participants. The differences were estimated with Friedman test (friedman, MATLAB) and pairwise Wilcoxon signed-rank tests (signrank, MATLAB). The results were corrected for multiple comparisons with Benjamini-Hochberg procedure (Benjamini & Hochberg, 1995;Groppe, 2024). Individual-level analysis revealed that many networks of the 19-network parcellation were not reproducible and, therefore, we excluded the 19-network parcellation from further analysis.

The Mantel test (Glerean et al., 2016) was used to test whether the interindividual variability of the parcellations reflects variability in the cortical thickness or curvature. The thickness and curvature maps for each participant were created using the FreeSurfer recon-all pipeline. The correspondence between the curvature maps and the 3D anatomy within STC is illustrated inFigure S3. Thereafter, we extracted the STC area used in the parcellations from the thickness and curvature maps of each participant and represented them as vectors. The similarity matrices were generated for the cortical curvature and thickness by computing pairwise Spearman correlations between participants using the vectors describing their STC thickness and curvature, respectively. A similarity matrix for the parcellations was created using pairwise Dice coefficients between individual-specific parcellations generated using all resting-state and task sessions. The Spearman correlation was computed between the upper triangle entries of the Dice similarity and thickness similarity matrices as well as between the Dice similarity and curvature similarity matrices. The statistical significance of the results was estimated by recalculating the correlations for 10,000 random permutations of the rows and columns of the Dice similarity matrix with respect to one another. The analysis was computed separately for each hemisphere and parcellation and corrected for multiple comparisons using Benjamini-Hochberg procedure with the significance threshold of 0.05 (Benjamini & Hochberg, 1995;Groppe, 2024).

#### 2.5.4 Comparing parcellations with brain areas activated by auditory or audiovisual stimulation

To estimate functional validity of the parcellations, we compared them to the brain areas activated during tasks. The activated brain areas were estimated with General linear model (GLM). GLM analysis was conducted with the fMRI analysis stream of Freesurfer, fs-fast, to estimate brain areas activated during auditory and audiovisual tasks. In the Tonotopy/AM task, the following contrasts were computed: (1) auditory stimulation vs. baseline, (2) high (1.87 kHz or 7.47 kHz) vs. low (0.12 kHz or 0.47 kHz) carrier frequencies, (3) slow (4 cycles/s) vs. fast (32 cycles/s) amplitude modulation, (4) slow amplitude modulation vs. baseline, and (5) fast amplitude modulation vs. baseline. The occasional target stimuli were excluded from all contrasts.

In the Audiovisual Speech/Noise task, we concentrated on three contrasts computed across the Auditory Clear / Visual Clear (AcVc), Auditory Clear / Auditory Noisy (AcVn), Auditory Noisy/ Visual Clear (AnVc), and Auditory Noisy/Visual Noisy (AnVn) stimuli. In main effect of Speech vs. Noise, we contrasted all possible audiovisual combinations with clear auditory signal with those with noisy auditory signal, i.e., ((AcVc+AcVn)-(AnVc+AnVn)) / 2. In the main effect of Lip motion vs. Noise, all audiovisual combinations with clear visual signal were contrasted with those with blurred vision, i.e., ((AcVc+AnVc)-(AcVn+AnVn)) / 2. We also calculated an audiovisual interaction that was specifically aimed at identifying the cortical areas where visual information of lip motion has the strongest effect of processing of speech sounds when the auditory signal is noisy. This contrast was, thus, defined as (AnVc-AnVn) - (AcVc-AcVn).

The group-level results were computed with a weighted least-squared random-effects model. If the task data were measured from the participant on 2 different days (Tonotopy/AM task for three participants, Audiovisual Speech/Noise task for seven participants), the measurements were combined with a fixed-effect model before computing the group-level results. The analysis was restricted to the auditory cortices (outlines shown inFigs. 5and6). The results were corrected for multiple comparisons with a cluster-wise Monte Carlo simulation (cluster-wise p-value threshold: p < 0.05, voxel-vise threshold: 1.3, sign: absolute, 10,000 simulations).

The outlines of the parcellations were overlaid on GLM task contrast maps at individual and group levels to visually estimate whether the parcellations align with the activated brain areas. Functional inhomogeneity was further used to evaluate the inhomogeneity of task activation in each network (Schaefer et al., 2018). Functional inhomogeneity of the parcellation was estimated by calculating the standard deviation in fMRI task activation (*z*-scores) within each network and averaging the standard deviations across networks while accounting for the network size:

$$\frac{\sum_{l=1}^{L}sd_{l}|l|}{\sum_{l=1}^{L}|l|}$$,

where$sd_{l}$is the standard deviation of task activation*z*-values for network*l*and|l|is the number of vertices in the network*l*(Schaefer et al., 2018).

The functional inhomogeneity was computed for each participant, using their task activation map with (1) their own individual-specific parcellation, (2) the group average parcellation, and (3) the individual-specific parcellations of all other participants (all parcellations were morphed to fsavreage6). The functional inhomogeneities between participants’ task activation and other participants’ parcellations were averaged across participants. The Friedman test (friedman, MATLAB) and pairwise Wilcoxon signed-rank tests (signrank, MATLAB) were used to estimate differences in the functional inhomogeneity between the three parcellations. The results were corrected for multiple comparisons with Benjamini-Hochberg procedure (Benjamini & Hochberg, 1995;Groppe, 2024).

Functional specificity of the STC parcellations was additionally evaluated for each task contrast by calculating the percentage of the total task effect each of the networks explained in each parcellations. This was done by dividing the sum of the GLM contrast effect size within each network by the sum of the GLM contrast effect size values within the whole parcellation. We also calculated Pearson correlation of these network-specific percentages of the total contrast effect size between tasks. This allowed us to evaluate whether the networks explaining most of the task effect differ between tasks.

### 2.6 Connectional homogeneity

The quality of the individual-level parcellations was further evaluated with connectional homogeneity of the resting-state data. If the individual parcellation captured actual functional organization of the individual’s STC, each network in the parcellation can be assumed to have homogeneous connectivity. To compute the connectional homogeneity for the parcellations, 12 resting-state fMRI runs were first concatenated within each participant. Second, pairwise Pearson correlation coefficients were computed between all vertices within each network of every participant. Third, these pairwise correlation coefficients were transformed into z-values using Fisher transformation. Finally, the z-values were averaged within each network, and thereafter, the averaged z-values were further averaged across all networks while accounting for network size:

$$\frac{\sum_{l=1}^{L}\;\rho_{l}|l|}{\sum_{l=1}^{L}|l|}$$,

where$\rho_{l}$is the connectional homogeneity for network*l*and|l|is the number of vertices in the network*l*(Schaefer et al., 2018).

The connectional homogeneity was computed for each participant, using their resting-state data and (1) their own individual-specific parcellation, (2) the group average parcellation, and (3) the individual-specific parcellations of all other participants (all parcellations were morphed to fsavreage6). The functional connectivity homogeneities between each participant’s data and other participants’ parcellations were averaged. The Friedman test (friedman, MATLAB) and pairwise Wilcoxon signed-rank tests (signrank, MATLAB) were used to determine whether the connectional homogeneity within networks differed between parcellations. The results were corrected for multiple comparisons with Benjamini-Hochberg procedure (Benjamini & Hochberg, 1995;Groppe, 2024).

### 2.7 Seed-based functional connectivity

Seed-based functional connectivity analysis was conducted to estimate with which cortical areas STC networks were connected. A seed-based connectivity map was generated for each network using that network as a seed region. To this end, the fMRI signals were averaged across vertices within the seed network as the seed signal. The functional connectivity map for the seed region was estimated in fsaverage6 surface by computing Pearson’s correlation between the seed signal and the fMRI signal from each cerebral cortical vertex of the same hemisphere. For the statistical analysis, the correlation values were normalized using Fisher*z*-transformation (Menon & Krishnamurthy, 2019). The statistical significance of the connectivity maps was estimated with a one-sample t-test (FreeSurfer, mri_glmfit). The statistical maps were corrected for multiple comparisons with the cluster extent correction using 5000 permutations and a voxel-wise cluster-forming threshold of 8. Further, we estimated pairwise differences between seed-based functional connectivity maps for adjacent networks in the 11-network parcellation using the Wilcoxon signed-rank test and Benjamini-Hochberg correction for multiple comparisons (Benjamini & Hochberg, 1995).

## 3 Results

### 3.1 Auditory cortex parcellations are reproducible within but highly variable across participants

**Fig. 2.**
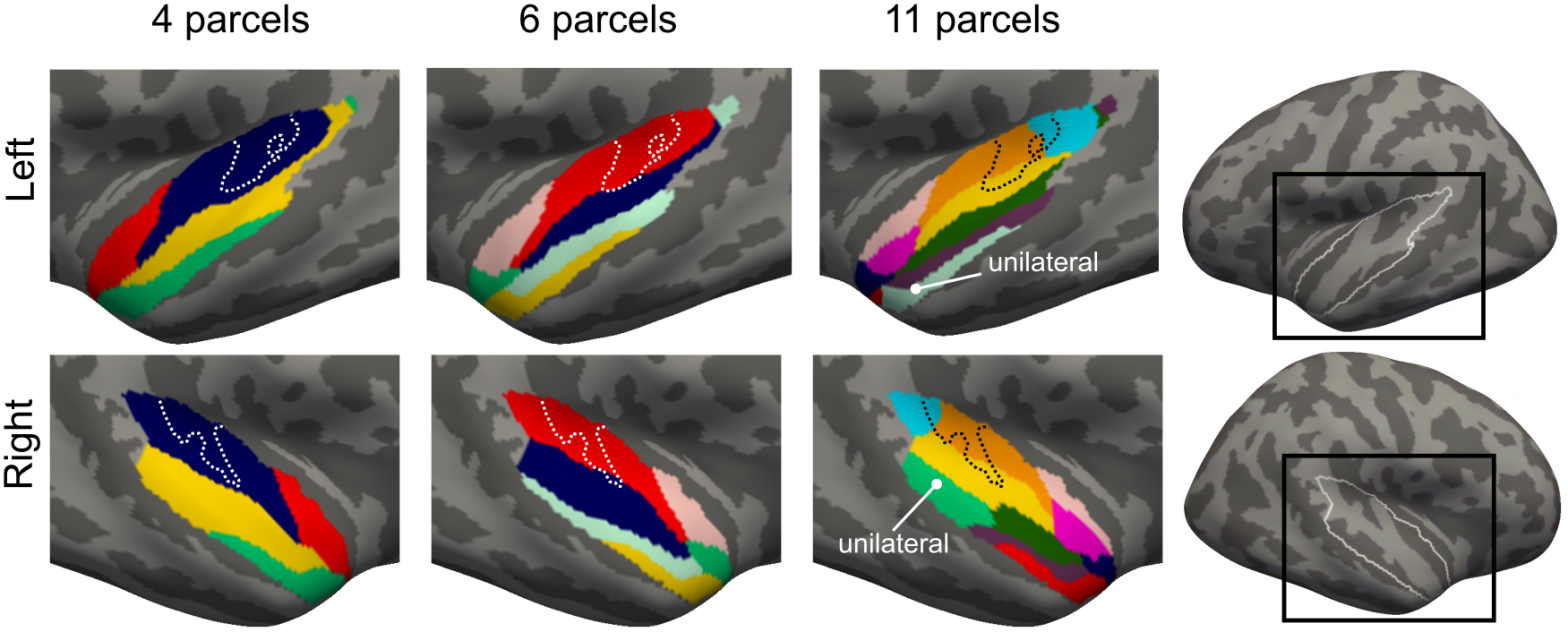
Group-level parcellations of auditory cortices in STC generated using an iterative functional network parcellation method. STC parcellations of 2–24 networks were investigated and three (4-, 6-, and 11-network parcellations) were selected for closer investigation since they yielded local maxima of Silhouette values and Dice coefficients at the group level. Different networks are marked with different colors. In the 11-network parcellation, 9 of the networks were bilateral and two unilateral. Heschl’s gyrus is marked with a dotted line.

We first generated 23 group parcellations based on functional connectivity by varying the number of clusters in K-means clustering from 2 to 24 (Fig. 1, steps 1–2). From these STC parcellations, 4-, 6-, and 11-network parcellations were selected for further analysis based on their group-level Silhouette and Dice coefficients (seeSection 2;Fig. 2andFigs. S2, S4, S5). To evaluate the test-retest reliability and intrasubject variability of the parcellations, we generated individual-level parcellations from each of the two resting-state sessions for each participant. For each session, the three STC parcellations were generated (containing 4, 6, and 11 networks). Thereafter, we calculated the overlap between parcellations generated from the two resting-state sessions, for each given cluster solution, by computing their Dice coefficient.

**Fig. 3.**
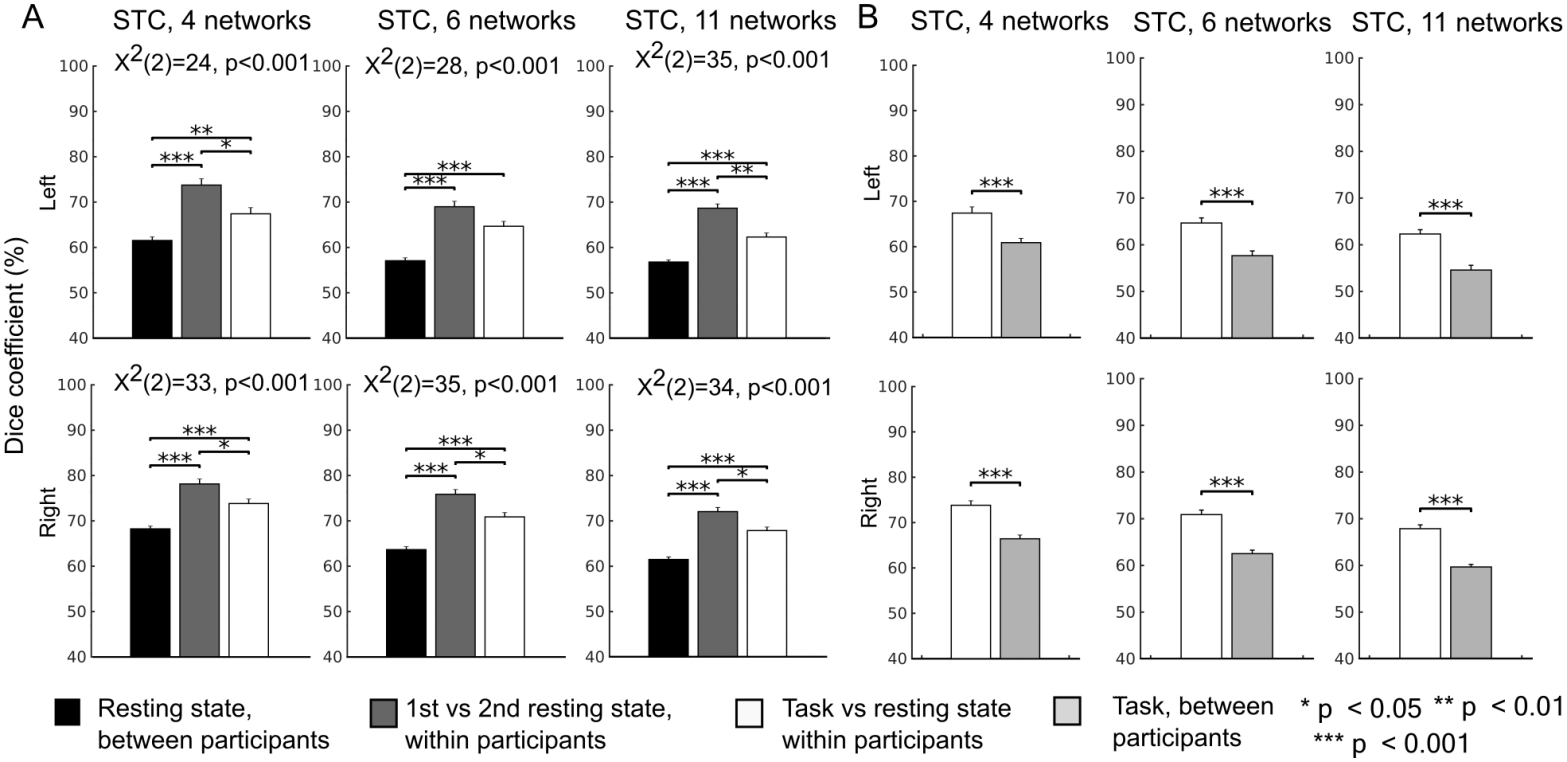
Intraindividual and interindividual variability of the parcellations. (A) The parcellations generated using the resting-state sessions demonstrated lower intraindividual than interindividual variability. When comparing two resting-state sessions of the same participant, on average, 69–78% of the STC vertices were assigned to the same networks. Between a given participant and any other participant, on average, only 57–68% of the STC vertices were assigned to the same networks. For each STC parcellation, the consistency of network membership was significantly higher within than between participants (p < 0.001 for all conditions). The overlap between the resting-state parcellations within participants was higher than that between the resting-state and task parcellations (task vs. rest: 62–74%, rest vs. rest: 69–78%, p < 0.05) for all parcellations expect the left hemispheric 6-network parcellation. This suggests that auditory cortex functional networks can be shaped by task. (B) The correspondence between task and resting-state parcellations of a given participant (62–74%) was higher than the correspondence between task parcellations of the participant and any other participant (55–66%; p < 0.001), suggesting than the parcellations are most similar within participants regardless of the task. The differences between these three conditions were estimated with the Friedman test and pairwise Wilcoxon signed-rank tests. The results were corrected for multiple comparisons using Benjamini-Hochberg procedure (Benjamini & Hochberg, 1995). Error bars indicate standard errors of the mean (SEM).

The parcellations were consistent within individuals between resting-state sessions (Figs. 3and4,Table S1). At the same time, the parcellations captured high interindividual variability within resting-state sessions. Notably, the interindividual variability was significantly higher than intraindividual variability (p < 0.001 for each parcellation, the Wilcoxon signed-rank test, corrected for multiple comparisons), indicating that the parcellations were robust and the interindividual variability was related to the actual functional organization of the brain rather than noise. Interindividual variability was significantly higher in the left than in the right hemisphere (4 networks: left: 62 ± 0.8%, right: 68 ± 0.6%; 6 networks: left: 57 ± 0.6%, right: 64 ± 0.6%; 11 networks: left: 57 ± 0.5%, right: 62 ± 0.6%, p < 0.001 for all parcellations). Again, each and every network was more consistent within than between individuals (Tables S4–S6), indicating that each network has substantial interindividual variability.

**Fig. 4.**
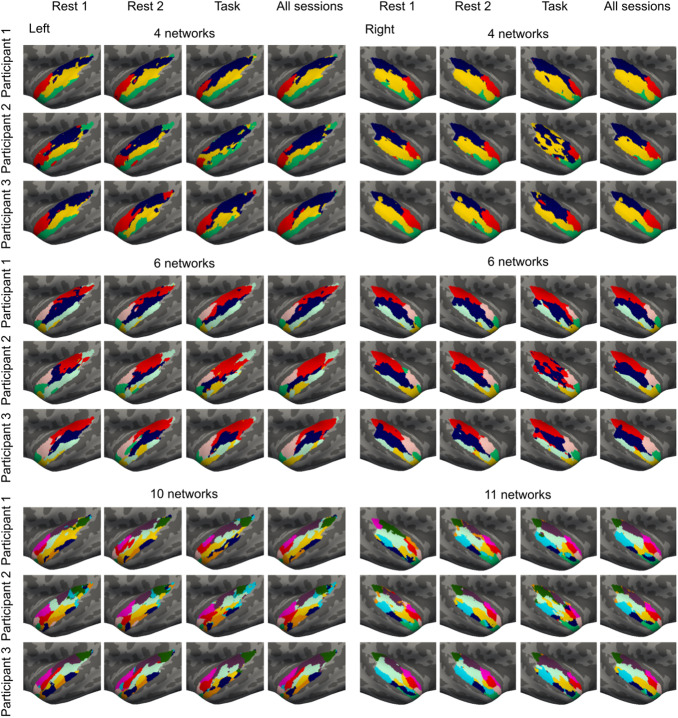
Individual-level 4-, 6-, and 11-network STC parcellations for three representative participants. The parcellations were generated separately for two resting-state fMRI sessions and for the session during which the participant was performing auditory and audiovisual tasks (seeSection 2for task descriptions). All sessions (i.e., Rest 1, Rest 2, and Task) were measured on different days. In addition, we generated parcellations using data from all three sessions.

To test whether the variability in the parcellations reflects variability in the cortical thickness and curvature, we conducted the Mantel test (Glerean et al., 2016) between the Dice coefficients and the STC thickness and curvature values. The surface representations of the STC thickness and curvature maps in the fsaverage6 were generated by FreeSurfer (Fig. S3). The surface representations allowed us to represent the individual cortical thickness and folding patterns as vectors. Thereafter, between-participant similarities for both of these anatomical measures were estimated with pairwise Spearman correlations between participants, resulting in one similarity matrix for cortical thickness and one for curvature. The between-participant similarity of the parcellations was estimated by creating a similarity matrix of the Dice coefficients between individual parcellations generated based on all resting-state and task sessions. According to the Mantel test, the matrix describing the parcellation similarity did not correlate with the matrices describing the similarity of the cortical thickness or curvature (r = -0.1–0.2, p = 0.38–0.86), suggesting that the interindividual variation is not affected by anatomical variability only.

### 3.2 Auditory cortex parcellations are most consistent within individuals regardless of the task

To investigate the reproducibility of the parcellations between different functional tasks, we compared the resting-state parcellations with the parcellations derived from the fMRI data measured during auditory and audiovisual speech processing tasks. STC parcellations were relatively consistent between resting-state and task sessions within participants (Fig. 3B,Table S2). Critically, the task parcellation overlapped more precisely with the resting-state parcellation of the same participant than with the task parcellations of the other participants (p < 0.001,Fig. 3B,Table S2): this suggests that the parcellations are most similar within individuals, independently of whether or not the participant was performing a task during scanning (Fig. 3B,Table S2). The overlap between task and resting-state parcellations was also higher within participants than the overlap of the resting-state parcellations between participants in all cases except in the right-hemispheric 4-network parcellation and the left hemispheric 6-network parcellation (p < 0.001,Fig. 3A). However, within participants, the two resting-state parcellations overlapped more precisely than resting-state and task parcellations (Fig. 3A,Table S3, p < 0.05) for all expect the left-hemispheric 6-network parcellation. This suggests that task can modulate functional networks of STC.

### 3.3 Task activations are more homogeneous within individual-specific than group parcellations

fMRI data collected during basic auditory stimulation and audiovisual speech processing were used to compare functional parcellations to brain responses elicited by tasks. Tonotopy and amplitude modulation rate representation were mapped orthogonally using the data from a task where the participants were presented with serial blocks of sounds, which varied both in their center frequency (tonotopy mapping) and in their amplitude modulation rate (rate representation mapping). InFigures 5and6, “Auditory vs. baseline” is the contrast between all auditory stimuli and no auditory stimulation, “High vs. low frequency” between high (1.87 kHz or 7.47 kHz) and low (0.12 kHz or 0.47 kHz) carrier frequencies, and “Slow vs. fast AM” between slow (4 cycles/s) and fast (32 cycles/s) amplitude modulations. The results for slow and fast AM rates versus baseline are shown inFigure S6. To localize the cortical areas responding to speech and audiovisual interaction, the participants were presented with clear or blurred video clips of a person voicing either “rain” or “rock.” The auditory component in the videos was either acoustically intact or replaced with noise that matched the spectrotemporal power distribution of the original speech. The “Speech vs. noise” contrast inFigures 5and6refers to the contrast between all conditions with clear speech and all with noise. “Visual input enhances noisy speech processing” refers to the areas where response to noisy speech was stronger when visual input was clear.

**Fig. 5.**
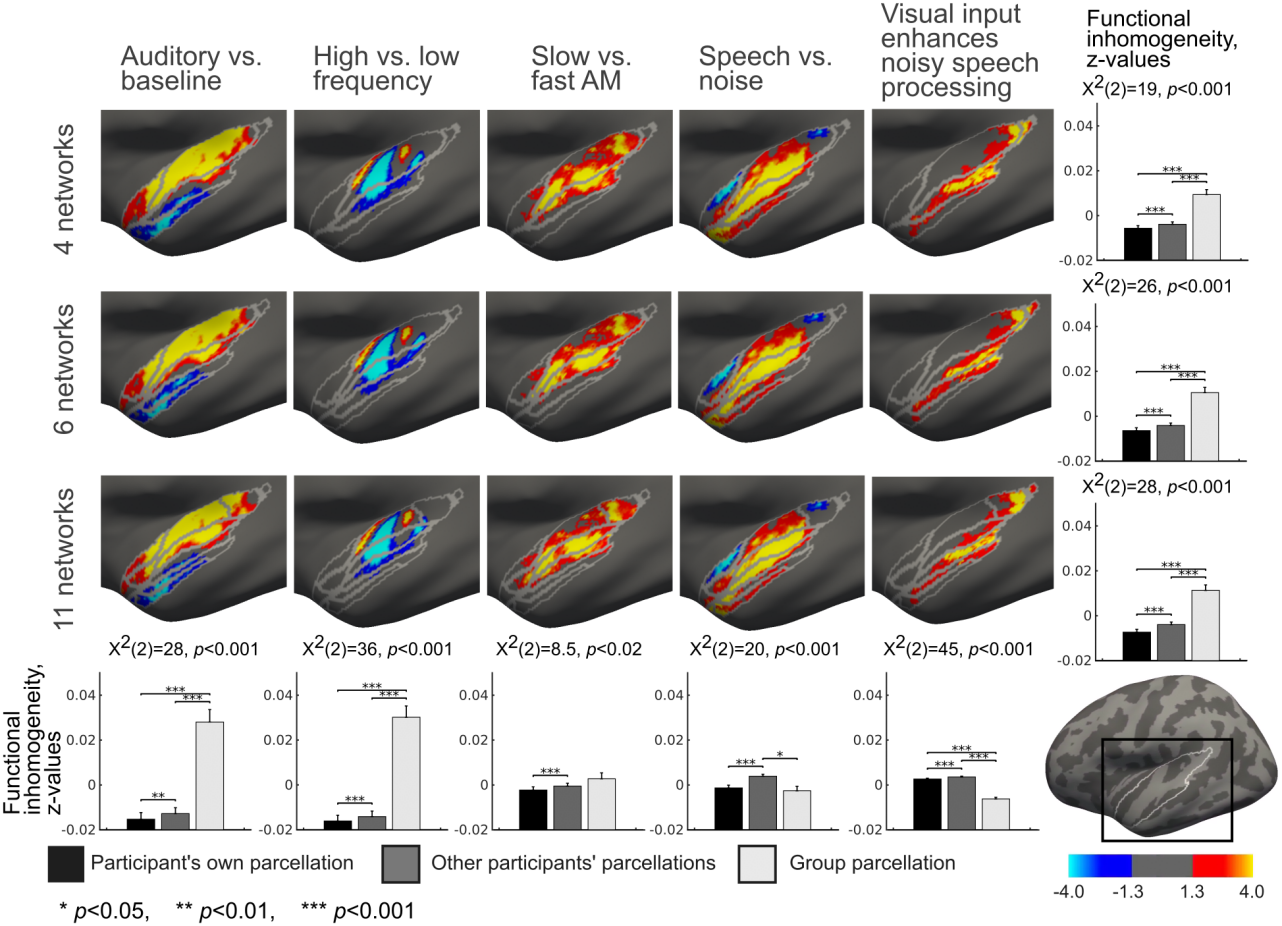
Topographic correspondence between the STC parcellations and GLM task contrasts in the left hemisphere. The group-level parcellation is overlaid on the group-level GLM contrasts computed from auditory and audiovisual tasks. The GLM analysis was computed within the auditory cortices. The bar diagrams in the right column show functional inhomogeneity averaged over all contrasts within each of the three parcellations. The bar diagrams in the bottom row show functional inhomogeneity averaged over all parcellations within each of the five contrasts. The functional inhomogeneity was computed between the individual GLM contrast map of each participant and (1) their own individual-specific parcellation, (2) individual-specific parcellation of all other participants, and (3) group-average parcellation. The differences between these three conditions were estimated with the Friedman test and pairwise Wilcoxon signed rank tests. The results were corrected for multiple comparisons using Benjamini-Hochberg procedure (Benjamini & Hochberg, 1995). Error bars indicate standard errors of the mean (SEM).

**Fig. 6.**
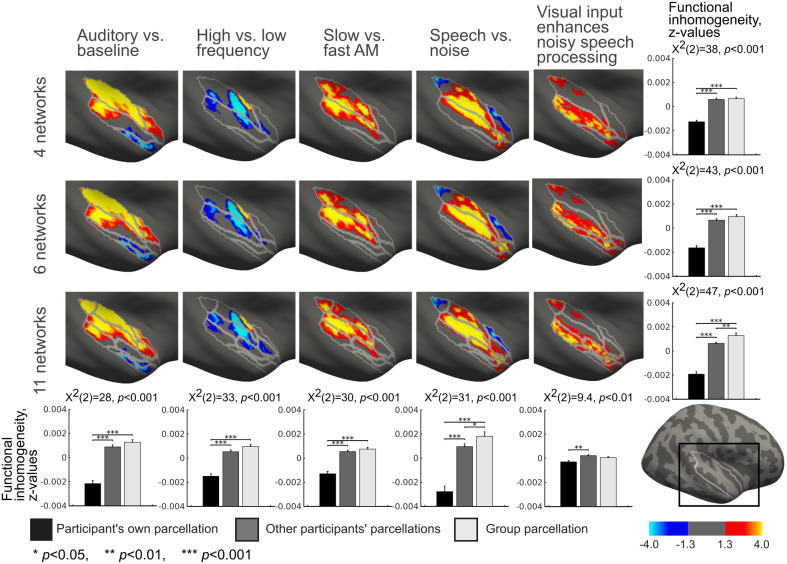
Topographic correspondence between the STC parcellations and GLM task contrasts in the right hemisphere. The group-level parcellation is overlaid on the group-level GLM contrasts computed from the auditory and audiovisual tasks. The GLM analysis was computed within the auditory cortices. The bar diagrams in the right column show functional inhomogeneity averaged over all contrasts within each of the three parcellations. The bar diagrams in the bottom row show functional inhomogeneity averaged over all parcellations within each of the five contrasts. The functional inhomogeneity was computed between the individual GLM contrast map of each participant and (1) their own individual-specific parcellation, (2) individual-specific parcellation of all other participants, and (3) group-average parcellation. The differences between these three conditions were estimated with the Friedman test and pairwise Wilcoxon signed-rank tests. The results were corrected for multiple comparisons using Benjamini-Hochberg procedure (Benjamini & Hochberg, 1995). Error bars indicate standard error of the mean (SEM).

If the parcellations represent functional areas, task response patterns can be expected to align with parcellation boundaries.Figures 5and6show the network parcellations overlaid with auditory and audiovisual speech fMRI task data. Based on visual inspection, the parcellation boundaries are reasonably well aligned with task response patterns. To quantitatively estimate the topographic correspondence between parcellations and task response patterns, we computed functional inhomogeneity for each parcellation. Functional inhomogeneity was defined as an average over network-specific standard deviations. The network-specific standard deviations were computed as the standard deviation of the task responses (*z*-scores) within each network, adjusted with the network size (seeSection 2). Low functional inhomogeneity indicates that parcellation aligns accurately with task response patterns. The functional inhomogeneity was estimated between each participant’s task response map and (1) individual-specific parcellation of the same participant, (2) individual-specific parcellations of the other participants, and (3) group-averaged parcellation (all parcellations were morphed to fsavreage6).

When averaged across five task contrasts, the individual-specific parcellation was functionally more homogeneous than other participants’ parcellations or group parcellation for all parcellations (p < 0.001,Figs. 5and6). This demonstrates the highest correspondence between individual-specific parcellation boundaries and individual task response patterns. When the functional inhomogeneities were averaged across the STC parcellations within contrasts, the individual-specific parcellation was functionally the most homogeneous (p < 0.01,Figs. 5and6,Fig. S6) for all except three contrasts in the left (Slow vs. fast AM, Speech vs. noise, Visual input enhances noisy speech processing) and one in the right hemisphere (Visual input enhances noisy speech processing). For “Slow vs. fast AM” contrast in the left and “Visual input enhances noisy speech processing” contrast in the right hemisphere, the participant’s own parcellation was functionally more homogeneous than other participants’ parcellations, but no statistical differences were found between the parcellations and the group-level parcellation. For “Speech vs. noise” contrast in the left hemisphere, no differences were found between the participant’s own and group parcellations but these both were functionally more homogeneous than other participants’ parcellations. For “Visual input enhances noisy speech processing” contrast in the left hemisphere, the group parcellation was functionally most homogeneous and the participant’s own parcellation more homogeneous than other participants’ parcellations.

**Fig. 7.**
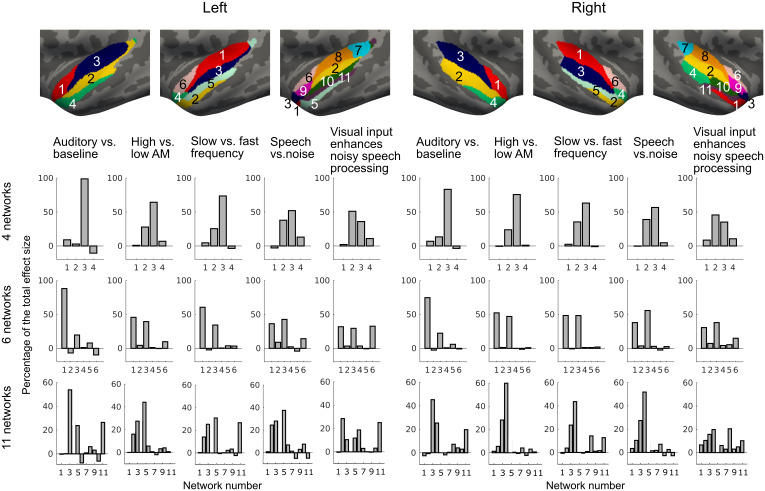
Functional specificity of the STC parcellations. The percentage of the total effect size is shown for each network in each parcellation. The task effect was concentrated within certain networks, suggesting functional specificity of the parcellations.

To further investigate the functional specificity of auditory areas in STC, we calculated the percentage of the total contrast effect size that each network of each parcellation explained.Figure 7shows that the task contrast effects were mostly restricted within certain networks, suggesting specificity of the STC networks. The correlation of the network-specific percentages of the total contrast effect size between task contrasts was lowest for the 11-network parcellation (left: 4 networks: 0.86, 6 networks: 0.84, 11-networks: 0.62; right: 4 networks: 0.90, 6 networks: 0.93, 11-networks: 0.83). This suggests that functional specificity was highest for the 11-network parcellation.

### 3.4 Connectional homogeneity is higher within individual-specific than group-parcellations

**Fig. 8.**
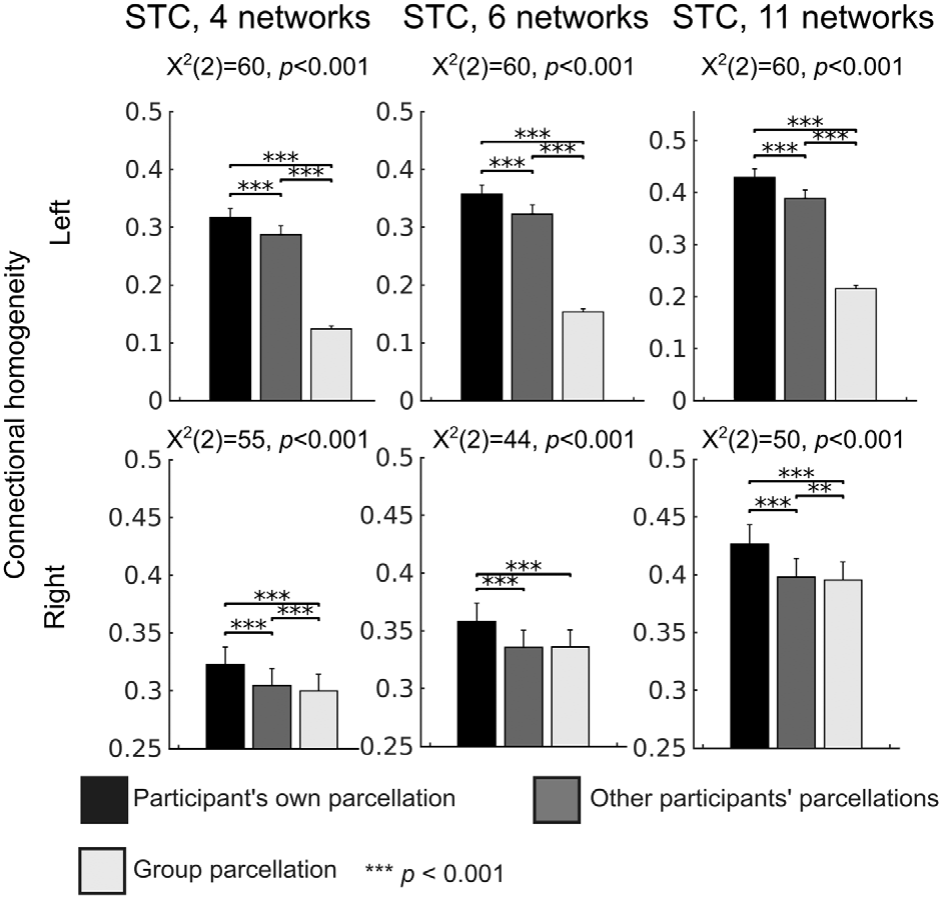
Connectional homogeneity of the STC parcellations. For each individual, connectional homogeneity was estimated using (1) their individual-specific parcellation, (2) the individual-specific parcellations of the other participants, and (3) the group-average parcellation. The statistical differences were estimated with the Friedman test and pairwise Wilcoxon signed-rank tests. The results were corrected for multiple comparisons with Benjamini-Hochberg procedure (Benjamini & Hochberg, 1995). The error bars indicate standard errors of the mean (SEM).

To further evaluate the neurophysiological plausibility and validity of the parcellations, connectional homogeneity was estimated using individual resting-state data. To estimate connectional homogeneity, Pearson correlation coefficients were calculated between all pairs of vertices within each network and transformed into z-values using Fisher z-transform. The resulting z-values were averaged within each network and, thereafter, an average was computed across network-wise z-values while accounting for the network size (seeSection 2). For each participant, the connectional homogeneity was estimated using their resting-state fMRI and (1) their individual-specific parcellation, (2) the individual-specific parcellations of the other participants, and (3) the group-average parcellation (all parcellations were morphed to fsavreage6). In this analysis, we used parcellations generated based on data from all three fMRI sessions. The connectional homogeneity was higher for the individual-specific STC parcellations than for other participants’ parcellations or for the group parcellation (Fig. 8, p < 0.001 for all parcellations), demonstrating that the individual-specific parcellations have the highest plausibility and validity. Further, the other participants’ parcellations yielded higher connectional homogeneity than the group-average parcellation (p < 0.01 for all parcellations) for all except the right-hemispheric 6-network parcellation for which no differences were found between these two parcellations.

### 3.5 Seed-based functional connectivity maps are specific to auditory cortex networks

**Fig. 9.**
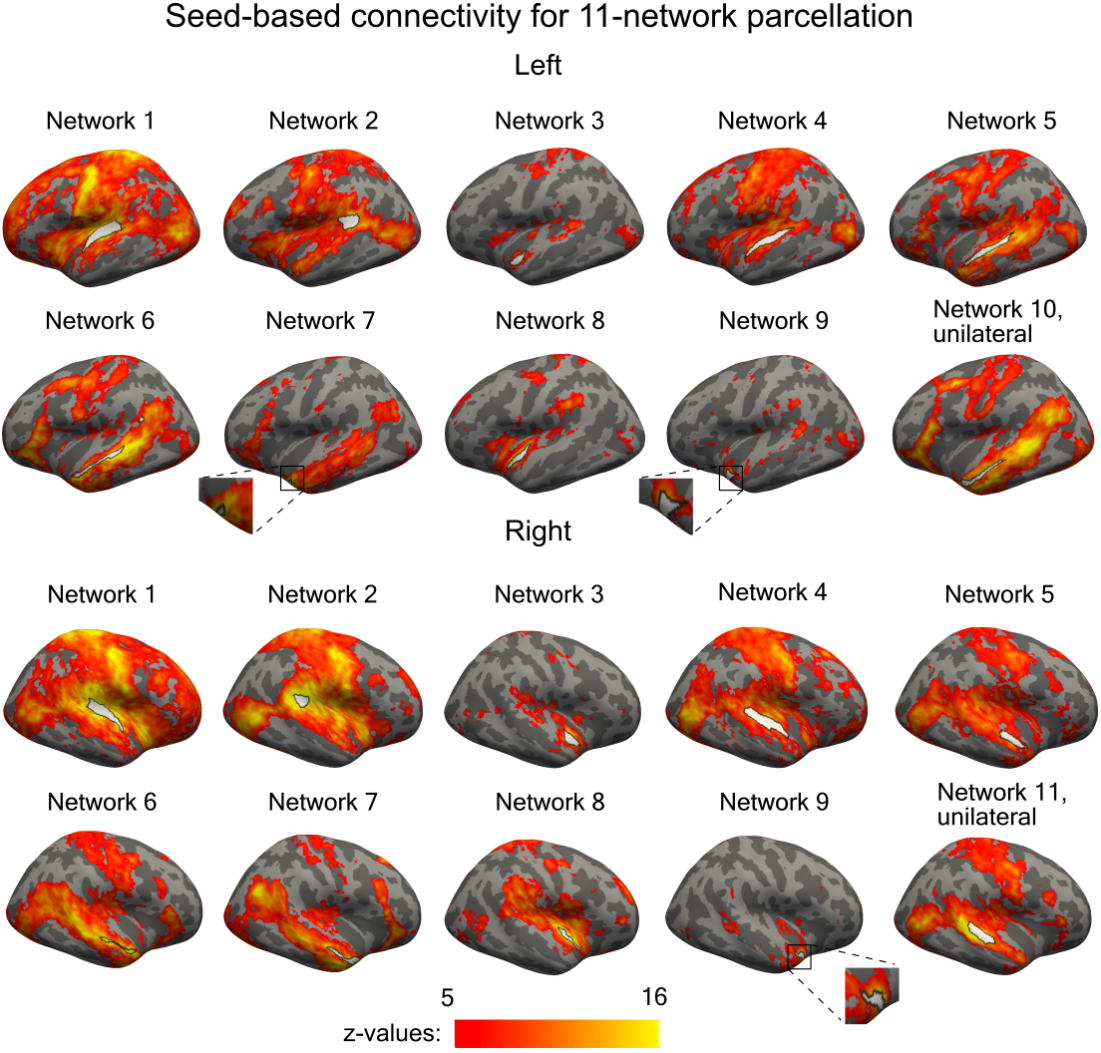
Seed-based functional connectivity maps for each network in the 11-network parcellation. Network 10 was only found in the left and Network 11 in the right hemisphere. Other networks were bilateral. One-sample t-test was performed for each vertex. The presented maps are thresholded at p < 0.05 and corrected for multiple comparisons with cluster-extent based permutation thresholding with a cluster-forming threshold of p < 0.001 (one-sample T-test).

**Fig. 10.**
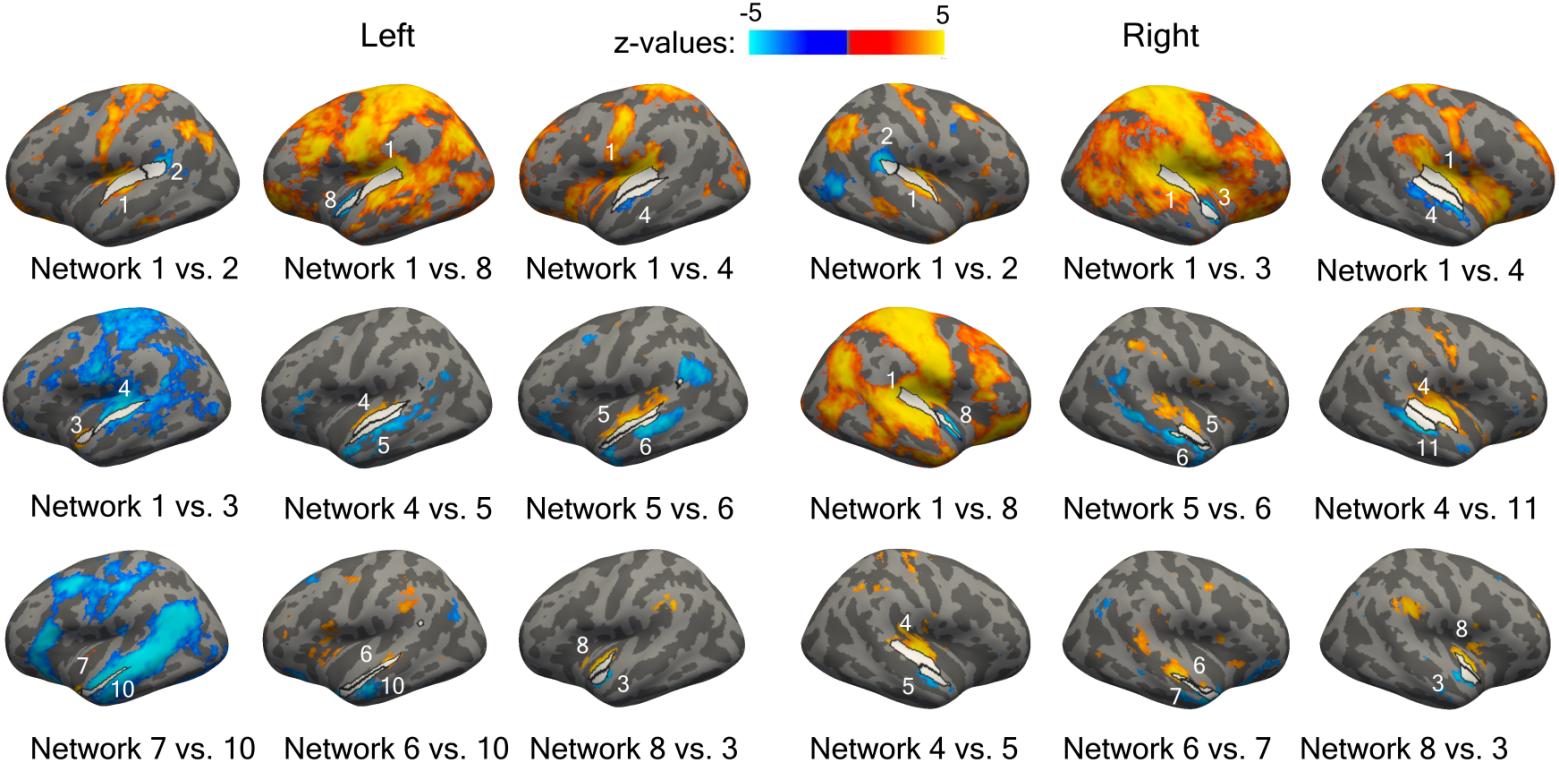
Differences between seed-based functional connectivity maps for pairs of adjacent networks in the 11-network parcellation. The Wilcoxon signed-rank test was calculated vertex wise between the connectivity maps of adjacent network pairs at each vertex. The resulting p-values were adjusted for multiple comparisons using Benjamini-Hochberg method (Benjamini & Hochberg, 1995). The threshold was set at p < 0.05.

The parcellation method classifies vertices with similar connectivity profiles into the same network. We conducted seed-based connectivity analysis using each network, in turn, as a seed region (an average signal was computed over the network vertices) to determine the cortical areas with which the STC networks were connected (Fig. 9;Figs. S7 and S8). As can be expected, the networks were connected with traditional speech and language areas, including temporal cortices, Broca’s area, and dorsal precentral areas (Hickok & Poeppel, 2004,^2007^). Some of the networks were also connected with lateral occipital/occipitotemporal cortices, including the human middle temporal area (hMT) that is associated with visuospatial motion processing (Bayerl & Neumann, 2002;Rokszin et al., 2010). The networks were also consistent with previous studies showing a dissociation between anterior/ventral “what” (Fig. 9, Networks 5-7, 10) and posterior/dorsal “where” pathways (Networks 2, 11;Ahveninen et al., 2013;Alain et al., 2001;Rauschecker & Tian, 2000). Moreover, the connectivity profiles of the adjacent STC networks showed statistically significant differences between each other even in the most detailed 11-network parcellation (Fig. 10).

## 4 Discussion

Our results suggest that human auditory areas in STC can be reliably divided into fine-grained subareas in individuals by using functional network analysis and ultra-high resolution 7T fMRI. The subarea arrangements were highly reproducible within individuals but showed substantial interindividual variability (*n*= 30). Moreover, the parcellations were functionally homogeneous and aligned with response patterns elicited by auditory and audiovisual tasks, which is indicative of their neurophysiological plausibility and validity. This is a notable extension to the auditory cortex parcellation literature, which has mainly focused on tonotopic mapping or group-level parcellations derived from anatomical data (for a group-level functional-connectivity based STC parcellation, seeRen, Hubbard, et al., 2021and for individual-level anatomical parcellation; seeLee et al., 2022).

### 4.1 The same auditory cortex subareas can be reliably identified in individuals at multiple network resolutions

Thus far, most existing parcellations of human auditory cortex have been derived from microanatomical architecture or using tonotopic mapping (for a review, seeMoerel et al., 2014). However, our recent study provided evidence that the human STC could be divided into subareas, at the group level, based on resting-state functional connectivity (Ren, Hubbard, et al., 2021). STC subareas have also been presented in the whole-brain resting-state functional connectivity-based parcellations (Chong et al., 2017;Glasser et al., 2016;Kong et al., 2021;Laumann et al., 2015;Yan et al., 2023). Studies using the large Human Connectome Project dataset have shown that the functional connectivity-based subareas of the temporal lobe roughly align with activations elicited by auditory and narrative (Glasser et al., 2016;Schaefer et al., 2018;Yan et al., 2023). The current study investigated the STC functional organization in more detail by generating and validating individual-level functional connectivity-based parcellations for STC at different network resolutions. Our study also includes auditory and audiovisual task and resting-state fMRI data from the same participants, which allowed us to evaluate the functional properties of the STC networks.

The 4-, 6-, and 11-network STC parcellations selected in the individual-level analysis were highly reliable within participants as indicated by a test-retest reliability of 69–78% for the resting-state data. Moreover, the parcellation boundaries were aligned with GLM topographies elicited by auditory and audiovisual tasks, which further supports their neurophysiological validity. Interestingly, the shape and size of the networks also differed between hemispheres, which is in line with the known hemispheric lateralization of the auditory areas.

While the 4- and 6-network parcellations were fully bilateral, the 11-network parcellation showed two auditory cortex subareas that were reliably present in only one hemisphere (Figs. 2and9). In the seed-based analysis, the left-hemispheric Network 10 was connected to the classic speech processing networks including Broca’s and dorsal precentral areas, consistent with the known lateralization of human language pathways (Hickok & Poeppel, 2004,^2007^). The right-hemispheric Network 11 was, in turn, connected to lateral occipital/occipitotemporal cortices, including the human middle temporal area (hMT) that is associated with visuospatial motion processing (Bayerl & Neumann, 2002;Rokszin et al., 2010). Network 11 could, thus, play a role in providing crossmodal visuospatial information to the auditory cortex, to support auditory motion processing. The lateralization of this network is consistent with previous studies on auditory motion processing (Baumgart et al., 1999).

Based on previous studies on humans and nonhuman primates, STC could include over 10 subareas within a limited region of the superior temporal cortex (Hackett et al., 2001;Kaas & Hackett, 2000;Kosaki et al., 1997;Merzenich & Brugge, 1973;Morel et al., 1993;Rauschecker & Tian, 2004;Rauschecker et al., 1995). Tonotopic mapping studies have typically identified three regions at the group level, but results in individuals suggest more fine-grained structure with additional frequency gradients (Moerel et al., 2014).Ren, Hubbard, et al. (2021)selected 2- and 6-cluster functional network-based parcellations for STC based on the Silhouette values. Further, several metrics and algorithms have been proposed for estimating the number of networks for connectivity-based parcellations (Eickhoff et al., 2015;Fraley & Raftery, 1998;Lange et al., 2004;Yeo et al., 2011). However, given the hierarchical multi-resolution organization of the human brain, the optimal number of networks may depend on the brain function of interest. Therefore, this study proposed three STC parcellations with network numbers of 4, 6, and 11. These parcellations showed local maxima of test-retest reliability and cluster separability among 2–23 network solutions, but other solutions may still be valid depending on the application, and the optimal number of networks may vary even between participants.

### 4.2 Parcellations reflect functional specificity of auditory cortex

The parcellation subarea outlines were well aligned with the GLM contrast topographies (Figs. 5and6). Many of the task-based contrast effects were largely concentrated within a few parcels only (Fig. 7). The 11-network parcellation appeared to align with the task contrasts most accurately. We also computed the percentage of total task effect size explained by each network. The correlation of these network-wise percentages between tasks was lowest for the 11-network parcellation. This suggests that the task determined more strongly for the 11-network parcellation within which networks the contrast effects were restricted than for the 4- or 6-network parcellations. Overall, the concentration of task-specific effects within selected parcels supports an interpretation that these parcels could reflect functionally relevant auditory subdivisions of STC.

The parcellation followed borders of the auditory processing hierarchy. The cochleotopic areas selective to high or low frequency were bilaterally restricted within the network (marked with orange inFig. 2), which included medial aspects of STC and early auditory areas of HG. The most significant responses to fast AM vs. baseline were also concentrated in this network. The two adjacent networks in the lateral (yellow) and posterior (turquoise) directions from the earliest areas responded more strongly to slow than fast AM, and also showed a strong contrast to auditory speech versus noise signals. These results agree with previous evidence that areas lateral to early auditory cortices integrate information over longer timescales and are more sensitive to complex than simple sound attributes (Cusinato et al., 2023;Norman-Haignere et al., 2022). However, in line withLeaver and Rauschecker (2016), no evidence for a clear large-scale amplitude modulation rate topography was found.

The area responding more strongly to speech than noise extended to two left-hemispheric networks in the lateral STG and the superior temporal sulcus (STS; dark violet and light turquoise). These networks showed no AM-rate or frequency selectivity, which is consistent with the notion that these areas respond more strongly to words than tones as well as to speech than non-speech sounds (Binder et al., 2000).

Within the most posterior (turquoise) and lateral (left: dark green, purple; right green, dark green) networks, the contrast between clear versus noisy visual stimulus was stronger when the accompanying auditory speech signal was noisy than when it was clear. In other words, in these subareas, visual gestures had the strongest effect on speech processing when the auditory signal is noisy. This may reflect networks that use multisensory information in challenging listening conditions. This finding is consistent with previous studies using the same stimuli in intracranial recordings in human participants, which reported that posterior areas of the non-primary auditory cortex are specifically important for multisensory integration of noisy speech signals (Ozker et al., 2017,^2018^).

### 4.3 Auditory cortex subareas vary substantially across individuals

The inherent significance of the functional variability of human auditory cortex has received relatively little attention. Here, we showed that fine-grained functional regions of auditory areas in STC vary substantially between individuals (Dice for resting state data: 57–68%). Notably, the parcellations were highly reproducible within individuals (Dice for resting-state data: 69–78%), which indicates that the parcellations were reliable and captured idiosyncratic features that are related to brain function rather than noise. Functional homogeneity analysis of the resting-state data provided further evidence for neurophysiological validity by showing that the individual-specific parcellation results in functionally more homogeneous networks than the group-level parcellation or the individual-level parcellations not specific to the participant of interest. Additionally, the individual-specific parcellation yielded more precise correspondence within each individual’s task response topography than with group-level parcellation or the parcellations of the other participants. This suggests that individual variability in parcellations corresponds with individual variability in task-evoked functional representations and, therefore, in the function of the auditory cortex. Our results are consistent with studies that have shown correspondences between task-evoked fMRI-response patterns and individual-specific whole-brain functional network parcellations (Gordon et al., 2017;Laumann et al., 2015;Schaefer et al., 2018;Tavor et al., 2016). The individual variability found in auditory cortex parcellations is also in line with a recent study that found intrinsic functional connectivity patterns of the human auditory cortex to be highly variable across individuals (Ren, Xu, et al., 2021).

The interindividual variability of the auditory cortex parcellations was larger in the left (Dice for resting-state data: 57–62%) than in the right hemisphere (Dice for resting-state data: 62–68%). This lateralization pattern is consistent with a recent fMRI study comparing individual variability of functional connectivity in humans and macaques (Ren, Xu, et al., 2021), as well as with earlier studies reporting high interindividual variability in the left STG activity during word repetition tasks (Burton et al., 2001). One potential interpretation is that the left auditory cortex is more individually variable due to its contribution to language related functions, which are shaped by our life-long experiences and exposure to language.

In this study, the data were aligned between participants in the surface space based on anatomical registration methods adapted to each participant’s individual folding patterns (Fischl et al., 1999). The folding patterns were aligned by minimizing the mean squared difference between the cortical convexity of the individual and the group template, adjusted by the variability of the convexity across subjects. Although this surface-based method largely removes macro-anatomical variability (Frost & Goebel, 2012;Hinds et al., 2008), the alignment is less accurate in the areas that are not strongly related to major sulci and gyri (Frost & Goebel, 2013;Gulban et al., 2020). Here, a potential concern is the variability of curvature patterns in and around the main anatomical landmark of auditory cortex, HG, which in different individuals could consist of from one to up to three parallel folds. To account for these potential biases, we therefore normalized each individual participant’s cortical convexity parameters, which reflect the three-dimensional shape of their STC, to the same fsaverage6 surface representation that was used for our functional parcellations (for six representative examples, seeFig. S3). Using these standard-space parameter representations, we then conducted the Mantel-test to determine whether the between-participant similarity in parcellations correlates with between-participant similarity in STC folding patterns. Based on these analyses, the between-participant similarity in the parcellations did not correlate with the between-participant similarity in macroanatomy within STC (r = -0.1–0.2, p > 0.38). This suggests that the individual variability in the parcellations is not explained by anatomical variability alone.

The individual-level functional connectivity-based mapping could provide a complementary method for aligning data across participants according to their functional areas instead of anatomical landmarks, to account for dissociations between functionally defined areas and anatomical landmarks (Amunts et al., 2000;Andrews et al., 1997;Cohen et al., 2008;Uylings et al., 2005). For example, a recent study shows that individual-level functional connectivity-based parcellations improve the group estimates of task activations and functional connectivity (Li et al., 2019), as well as associations between brain data and behavioral and clinical measures compared to group parcellations (Lauren et al., 2021;Li et al., 2019). These studies also suggest that individual-level functional parcellations could serve as alternatives to task-based functional localizers (Li et al., 2019). This could provide significant benefits in studies in which running multiple different task-based localizers would not be practical or possible.

### 4.4 Auditory cortex functional connectivity networks are shaped by tasks but remain consistent within individuals

The reproducibility between the rest- and task-based parcellations (Dice: 62–74%) was lower than the reproducibility between rest-based parcellations (Dice: 69–78%), suggesting that the task performance can shape the functional connections between brain areas. This result is not fully consistent with the previous study reporting no differences between task and resting-state whole-cortex parcellations (Wang et al., 2015). One reason for the contradicting results may be that we used more fine-grained parcellations and higher-resolution 7T fMRI data, allowing the detection of more subtle differences. However, the parcellations derived from task data were more similar with the parcellations generated from the same participant’s resting-state data than with the task-based parcellations of the other participants. Thus, the functional organization of the brain is still most consistent within individuals regardless of how the brain is engaged in a task during scanning. This finding is consistent with a previous study showing that individuals can be identified from a large group based on their functional connectivity profiles regardless of the task conditions (Finn et al., 2015).

### 4.5 Limitations and future directions

Several limitations should be noted when interpreting the results of this study. First, the number of networks was selected by relying on technical criteria and may not be physiologically meaningful for each individual. The number of networks can differ between individuals, particularly when studying patients who have undergone functional reorganizations. To overcome this issue, the parcellation algorithm could be initialized from a group-level atlas with a large number of networks (e.g., 40), followed by a gradual refinement of the number of subdivisions by combining regions with similar time courses (e.g., Pearson correlation r > 0.5). Second, the functional parcellation was based solely on STC-cortical connectivity. Given the converging evidence of the participation of subcortical structures (Felix et al., 2018;Pannese et al., 2015;Souffi et al., 2021) and cerebellum (Petacchi et al., 2005;Sens & de Almeida, 2007) in auditory processing, future work should incorporate STC-subcortical and STC-cerebellar connectivity into the parcellation. Third, the parcellations should still be validated with other modalities, such as electrophysiological measurements or cortical stimulation. Fourth, to understand the functional meaning of the networks, the parcellations outlines should be compared with task activation topographies of a more comprehensive set of auditory stimuli. Fifth, our results indicated high interindividual variability in the functional organization of the auditory cortex and showed that this variability reflects variability in the processing of auditory information in the brain. To understand more precisely how this interindividual variability is reflected in auditory function, hearing abilities, and speech comprehension, the results should be compared with behavioral data from hearing and speech comprehension tests. Finally, by using more sophisticated registration methods (Frost & Goebel, 2013;Gulban et al., 2020), it can be possible to further minimize the effect of inadequate registration of cortical surfaces on the individual variability of the parcellations.

## 5 Conclusions

Our results describe individual-level functional connectivity-based parcellations of human auditory areas in STC at three different network resolutions. These parcellations were more similar within participants than between participants, suggesting that the parcellations are relatively reliable within individual and show meaningful interindividual variability. Furthermore, the parcellation specific to the individual yielded higher alignment with task response topographies of the same individual than with the parcellations of the other participants or group-level parcellation. This suggests that individual variability in parcellations reflects individual variability in auditory function. Our results extend previous literature of auditory cortex parcellation which have mainly focused on tonotopic mapping or group-level parcellations, derived from anatomical data. The high interindividual variability of the parcellations suggests that novel strategies considering the individuality of functional arrangements of the auditory cortex could provide a way to facilitate future studies of human auditory cognition.

## Supplementary Material

Supplementary Material

## Data Availability

The data of the present study are available from the corresponding author upon request.
