# Supplementary material for: Individual connectivity-based parcellations reflect functional properties of human auditory cortex
Source: Imaging Neurosci (Camb). 2025 Feb 25;3:imag_a_00486. doi: 10.1162/imag_a_00486 (PMC12319797; doi:10.1162/imag_a_00486)
Supplement: Supplementary Material [file imag_a_00486-supp.pdf]

Supplementary material

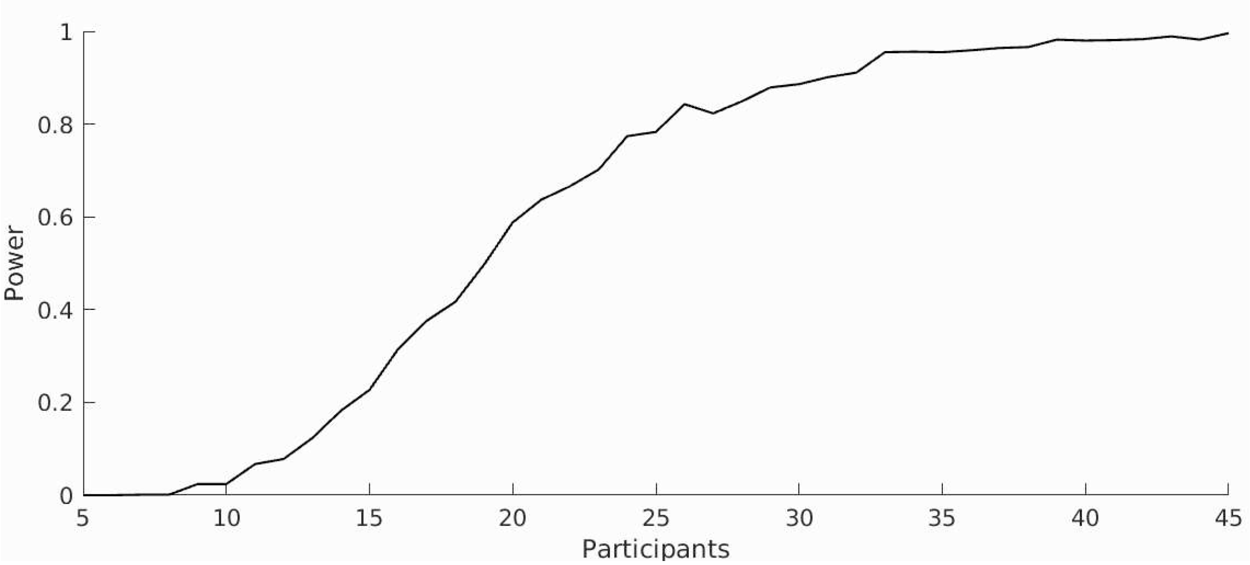

Figure S1. *Statistical power for detecting differences between intra- and interindividual variabilities as a function of the number of the participants. A power analysis was employed to confirm that the sample size of 30 participants used in this study was sufficient to detect the hypothesized effects (Kleinman & Huang, 2016). In the power analysis, we used bootstrapping to randomly select subsets of participants ranging from 5 to 45, with 1,000 iterations for each subset. For each iteration, all comparisons shown in Figure 3 were calculated and corrected for multiple comparisons. Based on a previously well-established procedure, the power was computed as a percentage of iterations in which all conditions were statistically significant (i.e., $p < 0.05$). Even with this conservative procedure, 30 participants achieved the statistical power of 0.9.*

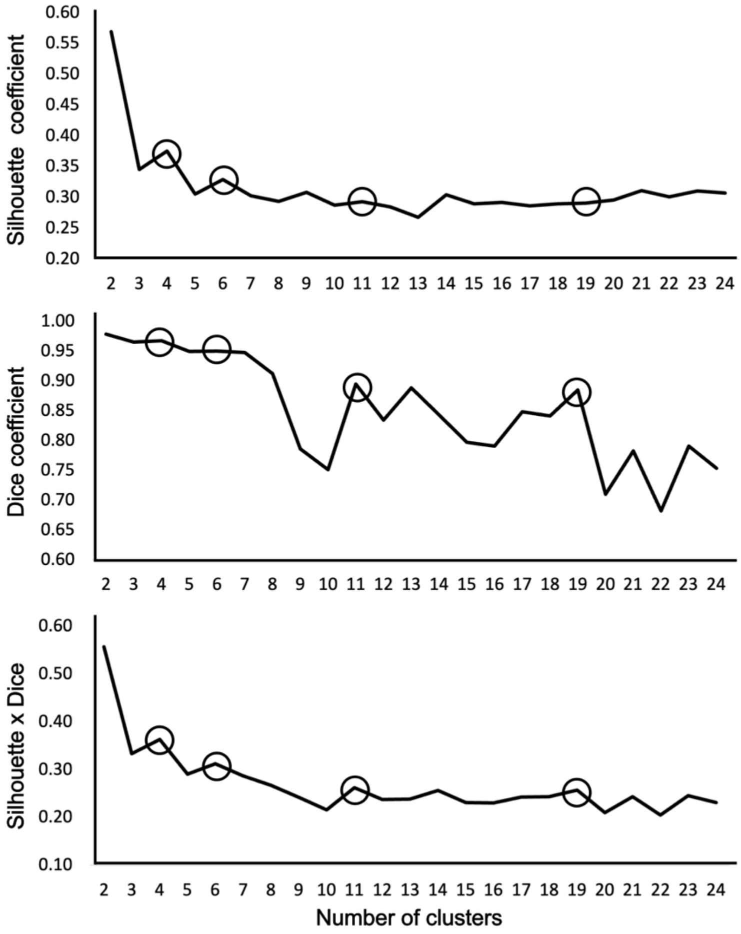

Figure S2. *Silhouette and Dice coefficients as well as their product computed for parcellations with network numbers from 2 to 24. The higher Silhouette coefficient means higher separability between clusters and the higher Dice coefficient higher reproducibility of the parcellations within participant. The parcellations selected for further investigation are marked with circles. The Dice coefficients are higher than reported in the other analyses because they were computed between parcellations derived from the first and last halves of runs whereas in the other analyses they were computed between parcellations derived from different sessions (see Methods).*

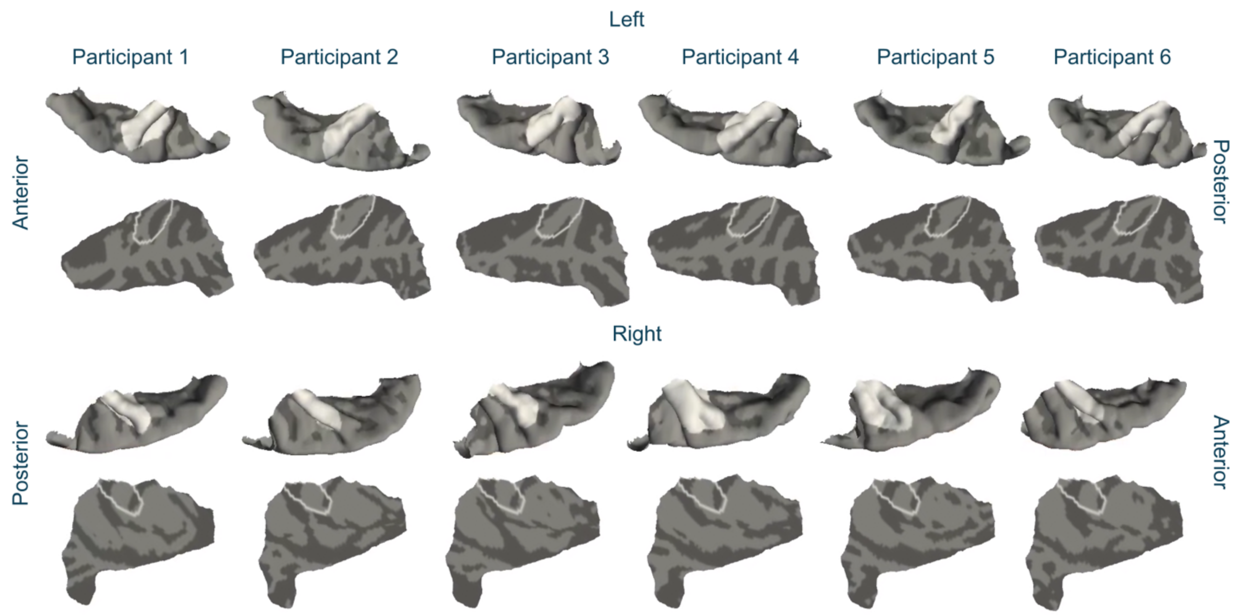

Figure S3. *3D STC anatomy and the corresponding flattened surface presentations in fsaverage6 for six participants of this study. The same STC areas that were used to generate the parcellations are shown. The curvature parameters are shown at a binary scale for clarity: dark gray refers to concave (i.e., sulcal) and light gray to convex (i.e., gyral) aspects of the cortex. Although the surface-based alignment of individual data to the template brain has been shown to effectively remove macro-anatomical variability of major sulci, there is still interindividual variability in the STC folding patterns mapped to fsaverage6 surface. However, the mapping of the folding patterns to the cortical surface allowed us to represent the folding patterns as a vector and quantitively estimate with Spearman correlation if the interindividual variability of the folding patterns explains the interindividual variability in the parcellations. The transversetemporal label generated by the recon-all pipeline is highlighted with white in the 3D presentations and outlined in the surface presentations.*

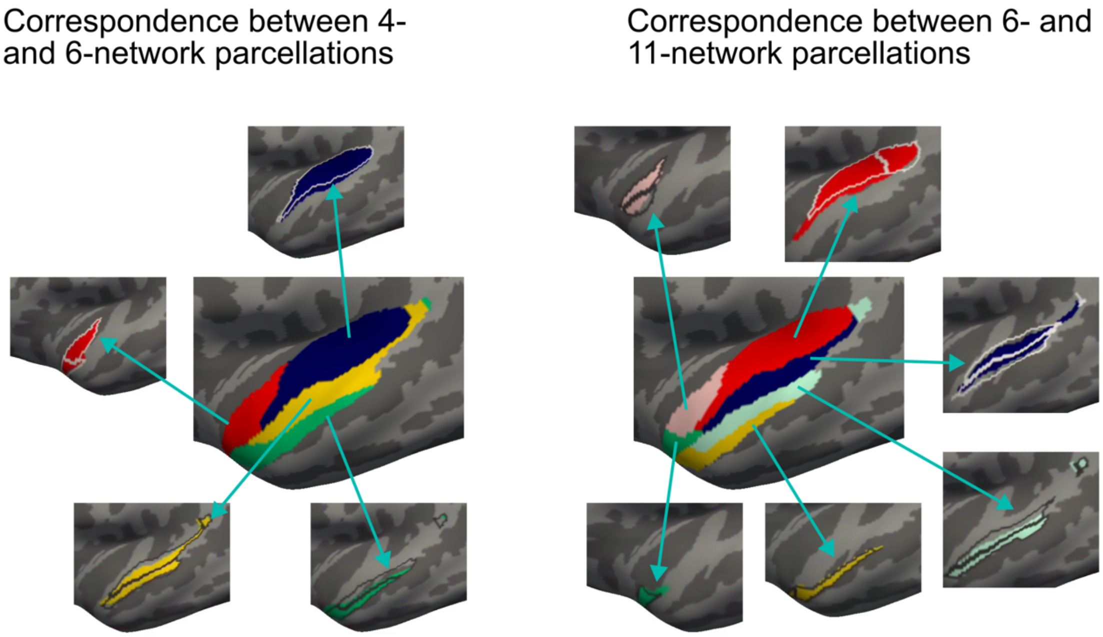

Figure S4. *Comparison of the individual networks between left-hemispheric parcellations. A) 4-network parcellation (middle) and each of its parcel in a separate window. The outlines of the corresponding parcels of the 6-network parcellation are shown on top of the individual parcels of the 4-network parcellation. B) 4-network parcellation (middle) and each of its parcel in a separate window. The outlines of the corresponding parcels of 6-network parcellation are shown on top of individual parcels.*

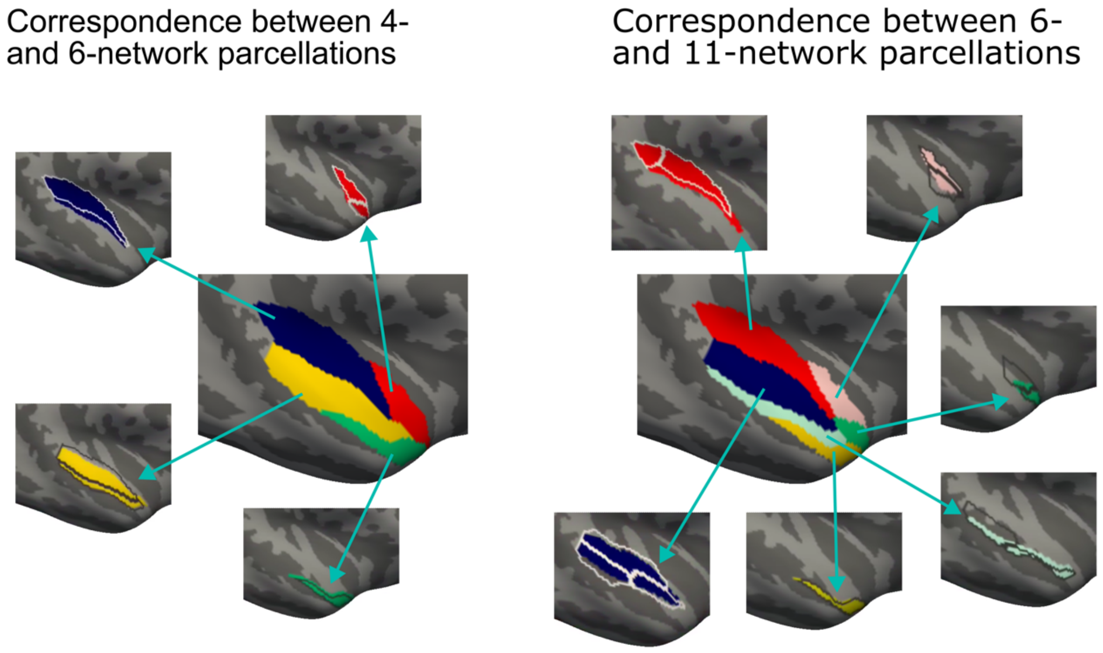

Figure S5. *Comparison of the individual networks between right-hemispheric parcellations. A) 4-network parcellation (middle) and each of its parcel in a separate window. The outlines of the corresponding parcels of the 6-network parcellation are shown on top of the individual parcels of the 4-network parcellation. B) 4-network parcellation (middle) and each of its parcel in a separate window. The outlines of the corresponding parcels of 6-network parcellation are shown on top of individual parcels.*

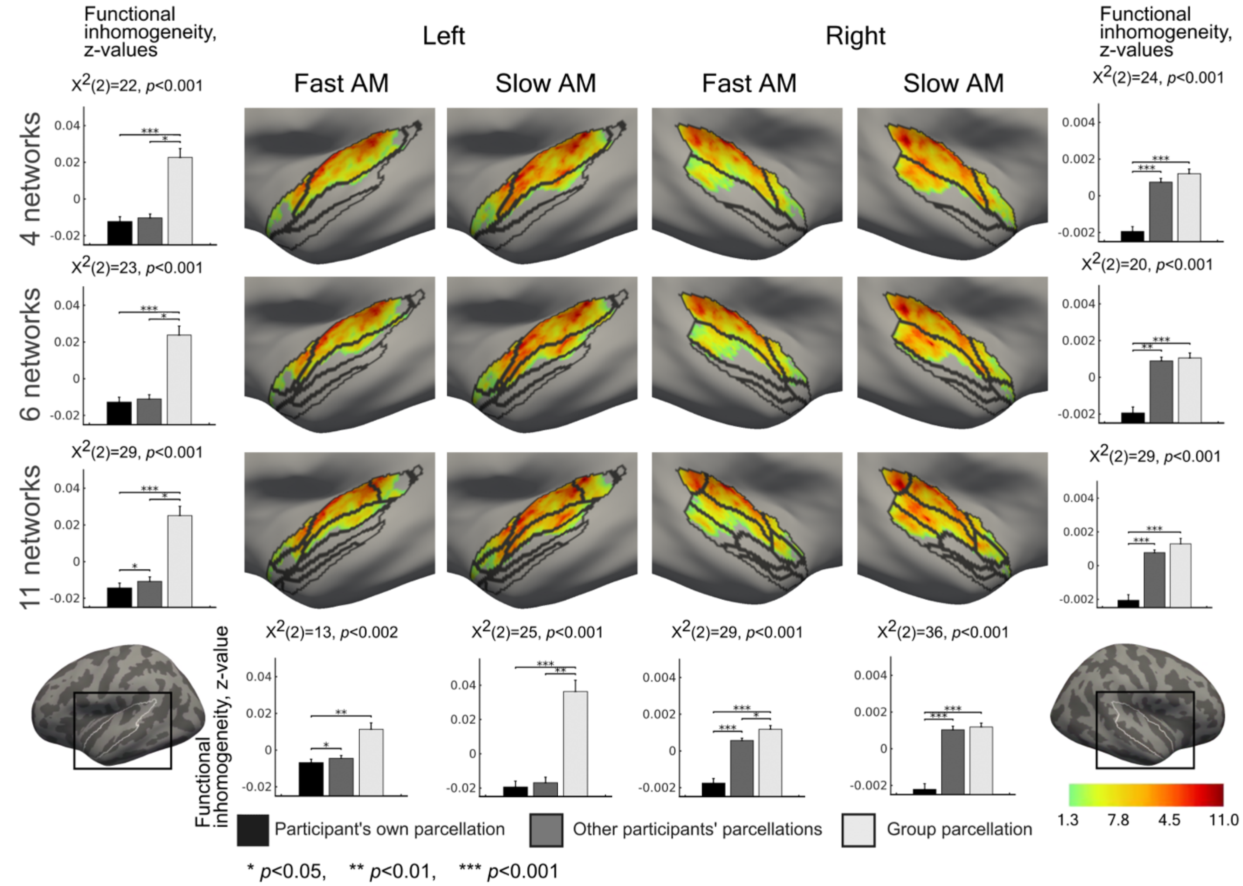

Figure S6. *Topographic correspondence between the STC parcellations and amplitude modulation rates, overlaid with the three studied group-level parcellations. The strongest activation to fast AM appears to concentrate to the more medial aspect of early auditory cortex than those for the slow AM. The bar diagrams in the right and left columns show right- and left-hemispheric functional inhomogeneity averaged over fast and slow AM contrast maps within each of the three parcellations. The bar diagrams in the bottom row show functional inhomogeneity averaged over all parcellations within each of the four contrasts. The functional inhomogeneity was computed between the individual GLM contrast map of each participant and 1) their own individual–specific parcellation, 2) individual-specific parcellation of all other participants, and 3) group-average parcellation. The differences between these three conditions were estimated with the Friedman test and pairwise Wilcoxon signed rank tests. The results were corrected for multiple comparisons using Benjamini-Hochberg procedure (Benjamini & Hochberg, 1995). Error bars indicate standard errors of the mean (SEM).*

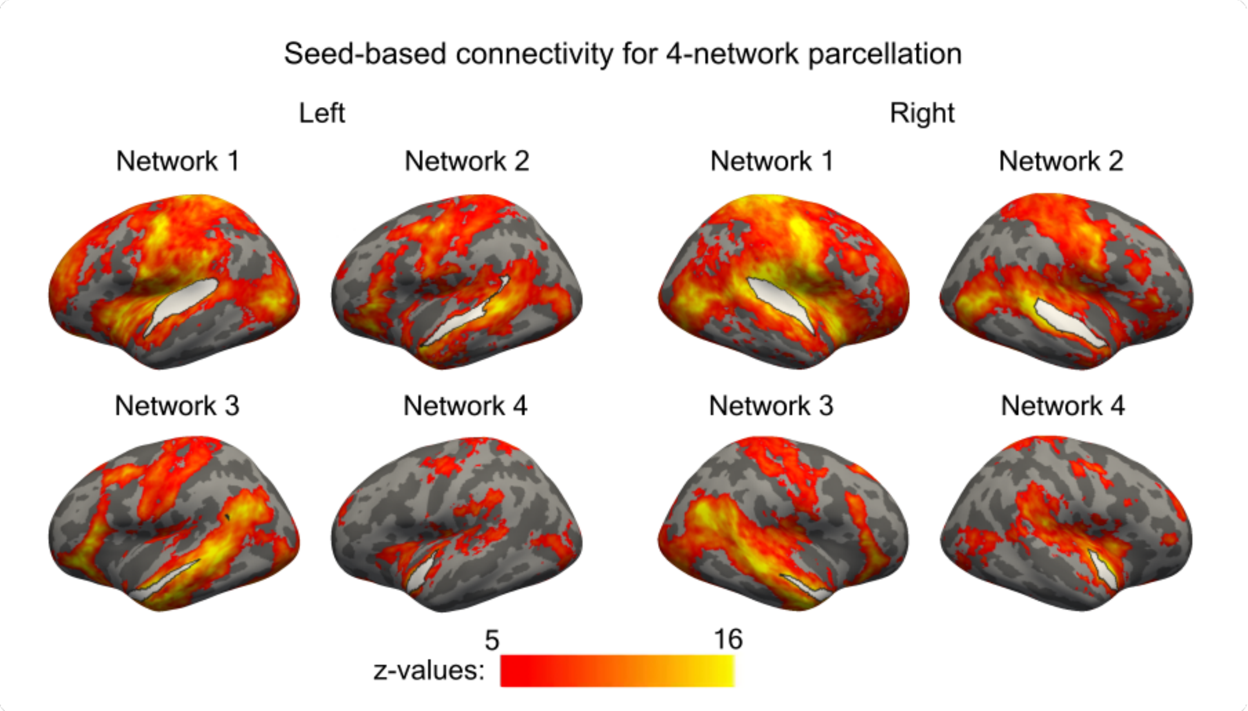

Figure S7. *Seed-based functional connectivity maps for each network in 4-network parcellation One-sample t-test was performed for each vertex. The presented maps are thresholded at p<0.05 and corrected for multiple comparisons with cluster-extent based permutation thresholding with a cluster-forming threshold of p<0.001 (one-sample T-test).*

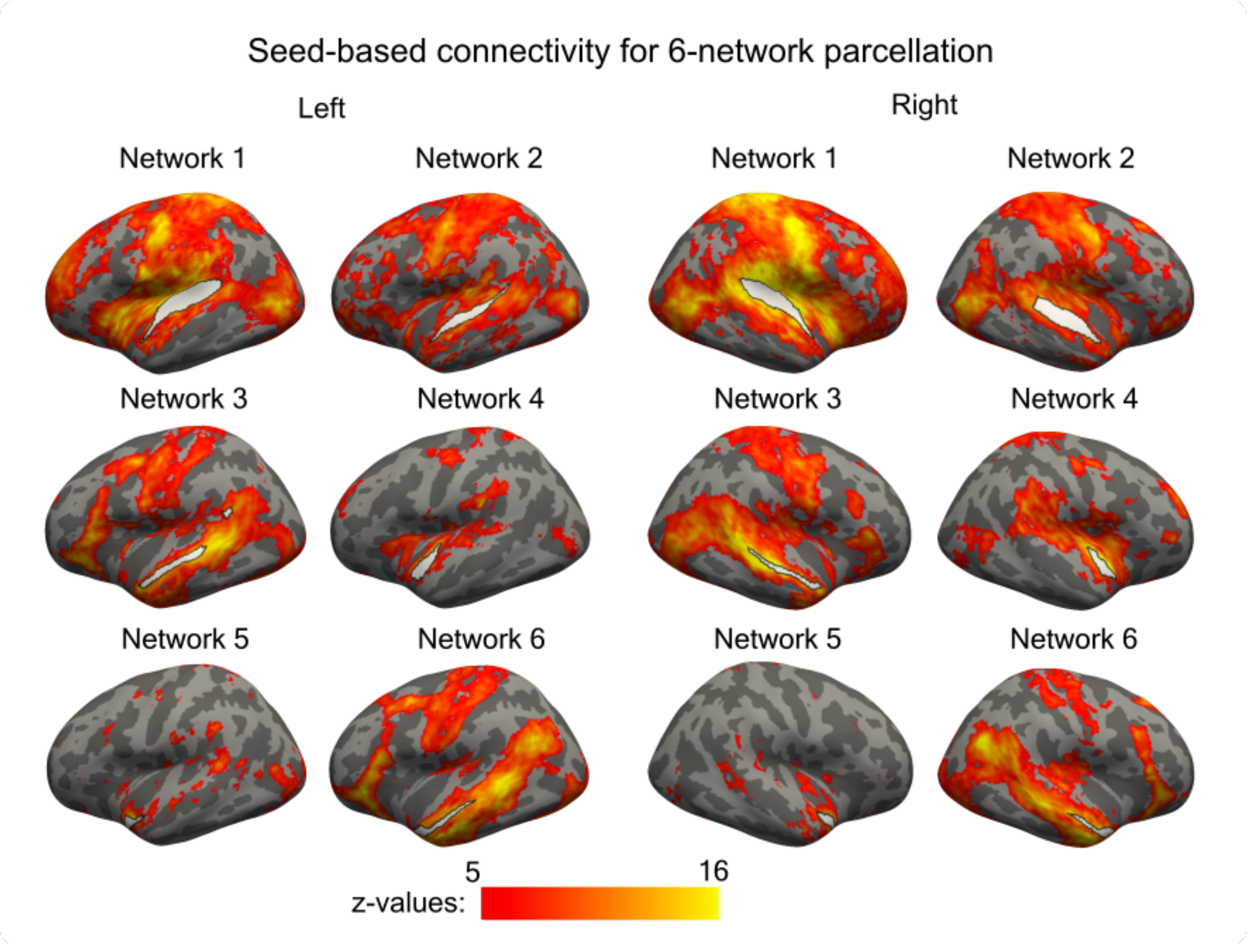

Figure S8. *Seed-based functional connectivity maps for each network in 6-network parcellation. One-sample t-test was performed for each vertex. The presented maps are thresholded at p<0.05 and corrected for multiple comparisons with cluster-extent based permutation thresholding with a cluster-forming threshold of p<0.001 (one-sample T-test).*

Table S1. *Dice coefficients (mean ± standard error of the mean, SEM) for resting-state parcellations within and between individuals.*

Number of networks	Hemisphere	Resting state, intraindividual (%)	Resting state, interindividual (%)	*p*-value
4	Left	74±1.4	62±0.8	<0.001
6	Left	69±1.2	57±0.6	<0.001
11	Left	69±1.0	57±0.5	<0.001
4	Right	78±1.1	68±0.6	<0.001
6	Right	76±1.1	64±0.6	<0.001
11	Right	72±0.9	62±0.6	<0.001

Table S2. *Dice coefficients (mean ± SEM) between resting-state and task parcellations within participants and task parcellations between participants.*

Number of networks	Hemisphere	Task vs. rest, intraindividual (%)	Task, interindividual (%)	p-value
4	Left	67±1.4	61±1.0	<0.002
6	Left	65±1.1	58±1.0	<0.001
11	Left	62±0.9	55±1.0	<0.001
4	Right	74±1.0	66±0.8	<0.001
6	Right	71±1.0	63±0.8	<0.001
11	Right	68±0.8	60±0.6	<0.001

Table S3. *Dice coefficients (mean ± SEM) between the two resting-state parcellations as well as between task and resting-state parcellations within individuals.*

Number of networks	Hemisphere	Resting state, intraindividual (%)	Task vs. rest intraindividual (%)	p-value
4	Left	74±1.4	67±1.4	<0.010
6	Left	69±1.2	65±1.1	<0.099
11	Left	69±1.0	62±0.9	<0.006
4	Right	78±1.1	74±1.0	<0.043
6	Right	76±1.1	71±1.0	<0.014
11	Right	72±0.9	68±0.8	<0.015

Table S4. *Dice coefficient (mean ± SEM) for each network of the 4-network parcellation. Intraindividual Dice coefficients were calculated between resting state parcellations created from the two resting state sessions of the same participant. Interindividual Dice coefficients were calculated between resting state-parcellations of different participants within the two sessions. The Dice coefficients were averaged over the sessions. P-value is the significance of the difference between the Dice coefficients (Wilcoxon signed rank test, corrected for multiple comparisons using Benjamini-Hochberg procedure across all parcellations and networks).*

Network	Hemisphere	Intraindividual (%)	Interindividual (%)	p-value
1	left	70±0.6	52±0.2	<0.001
2	left	77±0.4	64±0.2	<0.001
3	left	70±0.5	61±0.2	<0.001
4	left	79±0.4	68±0.2	<0.001
1	right	74±0.5	60±0.2	<0.001
2	right	78±0.3	70±0.1	<0.001
3	right	78±0.4	68±0.2	<0.001
4	right	84±0.3	74±0.2	<0.001

Table S5. *Dice coefficient (mean ± SEM) for each network of the 6-network parcellation. Intraindividual Dice coefficients were calculated between resting state parcellations created from the two resting state sessions of the same participant. Interindividual Dice coefficients were calculated between resting state-parcellations of different participants within the two sessions. The Dice coefficients were averaged over the sessions. P-value is the significance of the difference between the Dice coefficients (Wilcoxon signed rank test, corrected for multiple comparisons using Benjamini-Hochberg procedure across all parcellations and networks).*

Network	Hemisphere	Intraindividual (%)	Interindividual (%)	p-value
1	left	69±0.4	56±0.2	<0.001
2	left	68±0.4	60±0.2	<0.001
3	left	65±0.7	47±0.2	<0.001
4	left	74±0.4	66±0.2	<0.001
5	left	76±0.5	65±0.2	<0.001
6	left	63±0.6	49±0.2	<0.001
1	right	78±0.4	66±0.2	<0.001
2	right	77±0.4	69±0.2	<0.001
3	right	69±0.5	52±0.2	<0.001
4	right	76±0.5	64±0.2	<0.001
5	right	83±0.3	73±0.1	<0.001
6	right	73±0.5	58±0.2	<0.001

Table S6. *Dice coefficient (mean ± SEM) for each network of the 11-network parcellation. Intraindividual Dice coefficients were calculated between resting state parcellations created from the two resting state sessions of the same participant. Interindividual Dice coefficients were calculated between resting state-parcellations of different participants within the two sessions. The Dice coefficients were averaged over the sessions. Network 4 was rejected from the left and Network 3 from the right hemisphere and, therefore, they are not shown in the table. P-value is the significance of the difference between the Dice coefficients (Wilcoxon signed rank test, corrected for multiple comparisons using Benjamini-Hochberg procedure across all parcellations and networks).*

Network	Hemisphere	Intraindividual (%)	Interindividual (%)	p-value
1	left	60±0.5	45±0.2	<0.001
2	left	75±0.4	68±0.2	<0.001
3	left	65±0.6	53±0.2	<0.001
5	left	73±0.6	57±0.2	<0.001
6	left	65±0.7	52±0.3	<0.001
7	left	72±0.6	64±0.2	<0.012
8	left	61±0.6	44±0.2	<0.001
9	left	78±0.4	70±0.2	<0.019
10	left	76±0.3	67±0.2	<0.001
11	left	62±0.6	47±0.2	<0.001
1	right	66±0.6	55±0.2	<0.001
2	right	77±0.4	71±0.2	<0.036
4	right	77±0.4	63±0.2	<0.001
5	right	76±0.5	68±0.2	<0.001
6	right	66±0.6	58±0.3	<0.005
7	right	80±0.4	68±0.2	<0.001
8	right	63±0.6	48±0.2	<0.001
9	right	80±0.4	67±0.2	<0.001
10	right	74±0.5	69±0.2	<0.004
11	right	63±0.7	48±0.2	<0.001
